# miR-148a is Associated with Obesity and Modulates Adipocyte Differentiation of Mesenchymal Stem Cells through Wnt Signaling

**DOI:** 10.1038/srep09930

**Published:** 2015-05-22

**Authors:** Chunmei Shi, Min Zhang, Meiling Tong, Lei Yang, Lingxia Pang, Ling Chen, Guangfeng Xu, Xia Chi, Qin Hong, Yuhui Ni, Chenbo Ji, Xirong Guo

**Affiliations:** 1 Department of Children Health Care, Nanjing Maternity and Child Health Care Hospital Affiliated to Nanjing Medical University, Nanjing 210029, China; 2 Institute of Pediatrics, Nanjing Medical University, Nanjing 210029, China

## Abstract

Obesity results from numerous, interacting genetic, behavioral, and physiological factors. Adipogenesis is partially regulated by several adipocyte-selective microRNAs (miRNAs) and transcription factors that regulate proliferation and differentiation of human adipose-derived mesenchymal stem cells (hMSCs-Ad). In this study, we examined the roles of adipocyte-selective miRNAs in the differentiation of hMSCs-Ad to adipocytes. Results showed that the levels of miR-148a, miR-26b, miR-30, and miR-199a increased in differentiating hMSCs-Ad. Among these miRNAs, miR-148a exhibited significant effects on increasing PPRE luciferase activity (it represents PPAR-dependent transcription, a major factor in adipogenesis) than others. Furthermore, miR-148a expression levels increased in adipose tissues from obese people and mice fed high-fat diet. miR-148a acted by suppressing its target gene, Wnt1, an endogenous inhibitor of adipogenesis. Ectopic expression of miR-148a accelerated differentiation and partially rescued Wnt1-mediated inhibition of adipogenesis. Knockdown of miR-148a also inhibited adipogenesis. Analysis of the upstream region of miR-148a locus identified a 3 kb region containing a functional cAMP-response element-binding protein (CREB) required for miR-148a expression in hMSCs-Ad. The results suggest that miR-148a is a biomarker of obesity in human subjects and mouse model, which represents a CREB-modulated miRNA that acts to repress Wnt1, thereby promoting adipocyte differentiation.

Global prevalence of obesity in children, adolescents, and adults has significantly increased over the past decade and constitutes a growing public health crisis^1^. The combined prevalence of overweight and obesity combined (BMI ≥ 25) was over > 65% in the United States, and; from 1990–2000 to 2009–2010, the prevalence of grade 3 obesity (BMI ≥ 40) increased by 33%^2^. Obesity has also steadily increased in China. At present, approximately 21 million Chinese children are overweight; 50% of which are classified as obese^3^. Although this condition represents a major public health problem, many studies have not addressed the underlying useful therapeutic targets for obesity-associated metabolic syndrome. Thus, further understanding about the molecular mechanisms that initiate differentiation of stem cells into adipocytes in humans is necessary. At cellular level, increased adipose tissue mass is ascribed to the proliferation and hypertrophy of adipocytes^4^, with degree of hypertrophy relative to hyperplasia, influencing the percentage of body fat and the metabolic consequences of obesity^5^. Adipocyte differentiation is a highly regulated process that involves sequential activation of several transcription factors, including CEBPα, CEBPβ, and PPARγ^6^, as well as cAMP-response element-binding protein (CREB), which has been implicated as an early regulator of the adipocyte differentiation transcriptional program^7^. The activities of these transcription factors are partially coordinated by Wnt signaling, which is involved in self-renewal and differentiation of stem cells^8^. Wnt signaling was first recognized as a possible negative regulator of adipogenesis when Wnt1 expression decreased significantly during adipocyte differentiation progress^9^.

Human adipose-derived mesenchymal stem cells (hMSCs-Ad) represent a population of self-renewing and multipotent cells that differentiate into adipocytes and play an important role in adipose tissue hyperplasia^8^,^10^. Given the molecular pathways in this process are incompletely elucidated, investigation of the mechanism of adipocyte differentiation in hMSCs-Ad may provide better understanding of the pathogenesis of metabolic diseases, such as obesity and diabetes. As a step to identifying factors that modulate this process, we examined the roles of microRNAs (miRNAs) in adipogenesis because of their functions in a tissue- and cell type-specific manner, as well as their essential roles in many biological processes, including differentiation, proliferation, apoptosis, and development^11^. miR-125b, miR-22, miR-21, and miR-196a maintain the balance between adipocyte and osteogenic differentiation in hMSCs-Ad^12^,^13^,^14^,^15^,^16^, whereas miR-8^17^ and miR-143^18^ positively and miR-27a/b^19^,^20^ and let-7^21^ negatively regulate adipogenesis. miRNAs are also useful as disease biomarkers and therapeutic targets because of their stability^22^. To date, few key miRNAs controlling hMSCs-Ad differentiation into adipocytes have been identified^18^,^19^,^20^,^21^. However, the mechanism of new obesity-specific miRNA in this process has not been definitively linked to specific aspects of the hMSCs-Ad differentiation program and transcription factors that regulate miRNA transcription and adipogenesis.

In this study, miR-148a, miR-26b, miR-30, and miR-199a levels were increased in differentiating hMSCs-Ad. Among these miRNAs, miR-148a exhibited significant effects on increasing luciferase activity of PPRE, representing PPAR-dependent transcription, as a major factor in adipogenesis. Moreover, miR-148a was upregulated robustly in differentiated hMSCs-Ad, and its expression gradually increased during hMSCs-Ad differentiation. miR-148a directly bound to its target gene, Wnt1, to repress its expression. In addition, a major CREB was identified in the promoter sequence of miR-148a that regulated its expression. A positive correlation between adiposity and miR-148a expression was observed in obese mice, as well as in overweight and obese human subjects. These results establish a new role for miR-148a in regulating hMSC-Ad differentiation, thereby providing new insights into the processes that regulate obesity.

## Results

### miRNA expression profile in adipocytes

**Figure 1:**
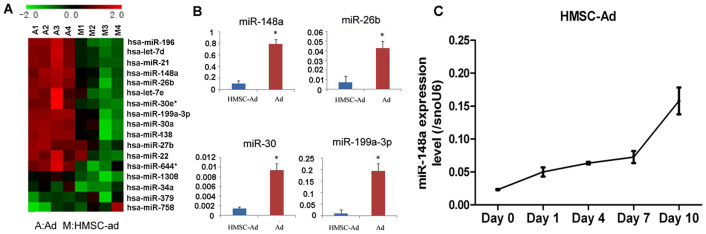
miRNA expression profile during adipogenesis in hMSCs-Ad. (A) Heatmap of the fold-changes (log2 transformed) of miRNA expression in differentiated vs. undifferentiated hMSCs-Ad cells. Differentially expressed miRNAs (*P* < 0.05) are shown. Each group (A1-A4 and M1-M4) is pooled from three samples. (B) miRNA profile analysis was performed by TaqMan miRNA kits. Ratios were calculated as mean ± SEM from triplicate samples. Data shown are averages of three independent experiments and were analyzed using Student’s *t*-test (paired, two-tailed). (C) hMSCs-Ad were grown to confluence, and adipogenic differentiation was initiated as described in Research Design and Methods. Expression of miR-148a during hMSC-Ad differentiation was quantitated by TaqMan miR-based qRT-PCR. Data shown are mean ± SEM of four independent experiments analyzed using one-way ANOVA (* *P* < 0.001) vs. undifferentiated hMSC-Ad on Day 0. Data shown are representative of three similar experiments.

To identify the miRNAs related to adipogenesis, hMSCs-Ad and adipocytes were analyzed for miRNA expression by miRNA microarray, and more miRNAs changed by at least twofold (*P* < 0.01) (Fig. 1A; partial data of microarray). miR-148a and miR-26b were highly expressed in differentiated hMSCs-Ad by over fivefold compared with undifferentiated hMSCs-Ad. miR-30 and miR-199a-3p were also highly expressed threefold in differentiated hMSCs-Ad compared with undifferentiated hMSCs-Ad. Changes in miR-148a, miR-26b, miR-30, and miR-199a-3p were confirmed by qRT-PCR (Fig. 1B), and these findings closely mirrored the array data. We then examined the role of these differentially expressed miRNAs in adipocyte differentiation by PPREx3-TK report gene, representing PPAR-dependent transcription as a major factor in adipogenesis^23^. miR-148a increased PPREx3-TK activity by about twofold (Fig. S1A). However, other miRNAs slightly increased the activity of PPREx3-TK. Thus, miR-148a may play a critical role in adipocyte differentiation and be identified as the robust candidate miRNA for further research. miR-148a was also upregulated sixfold in hMSCs-Ad. miR-148a expression gradually increased after induction of adipocyte differentiation in hMSCs-Ad and peaked after 10 d (Fig. 1C). miR-148a expression levels also increased 10-fold in murine 3T3-L1 preadipocytes undergoing differentiation (Fig. S1B), further suggesting that miR-148a is associated with adipocyte differentiation.

### Overexpression of miR-148a promotes adipogenesis in hMSCs-Ad

**Figure 2:**
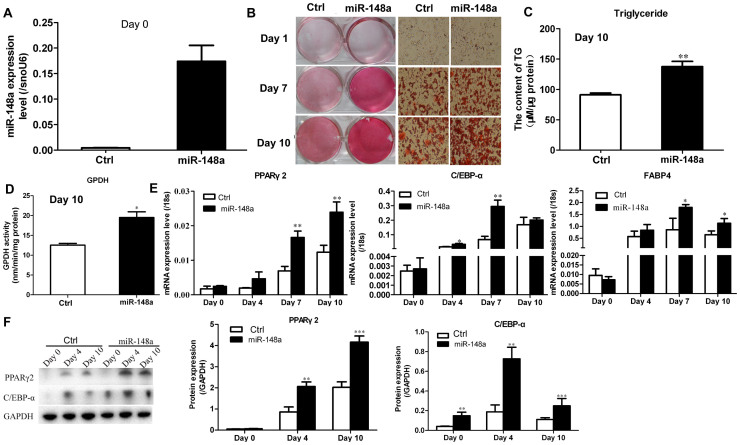
Overexpression of miR-148a in hMSCs-Ad enhances adipogenic differentiation. (A) miR-148a overexpression efficiency was verified by qRT-PCR at Day 0. (B) Oil red O staining indicated the effect of miR-148a overexpression on hMSCs-Ad adipogenic differentiation at Days 1, 7, and 10. (C) Triacylglycerol content and GPDH activity (D) detected neutral lipid accumulation. (E) miR-148a overexpression in hMSCs-Ad promoted adipogenic maker gene transcription based on qRT-PCR at Days 0, 4, 7, and 10 during adipogenesis. (F) PPARγ2 and C/EBP-α protein levels were detected by Western blot under the same experimental conditions. Related mRNA levels are represented as mean ± SEM from three or more independent repeats. *P < 0.05, **P < 0.01, ***P < 0.001.

To determine whether miR-148a directly affected adipocyte differentiation, hMSCs-Ad were transduced with a lentivirus expressing miR-148a or an empty virus for 48 h before transferring to differentiation medium. After selection, > 95% of cells were measured GFP-positive by fluorescent microscopy, and transduced hMSCs-Ad did not exhibit morphological changes compared with control (Fig. S2A). The expression of miR-148a was increased 38-fold at Day 0 and remained high throughout differentiation (Fig. 2A). As control, the expression of miR-152, which belongs to the same family of miR-148a, as well as miR-1908 and let-7a, was unchanged (Fig. S2B), suggesting that miR-148a did not disrupt other endogenous miRNA pathways. Overexpression of miR-148a promoted adipogenesis as indicated by oil red O staining (Fig. 2B), triacylglycerol content (Fig. 2C), and GPDH activity (Fig. 2D). Additionally, qRT-PCR (Fig. 2E) and Western blot (Fig. 2F) analyses indicated that the adipocyte-specific factors PPARγ2, C/EBP-α, and FABP4 increased significantly after transduction with the miR-148a-expressing lentivirus.

**Figure 3:**
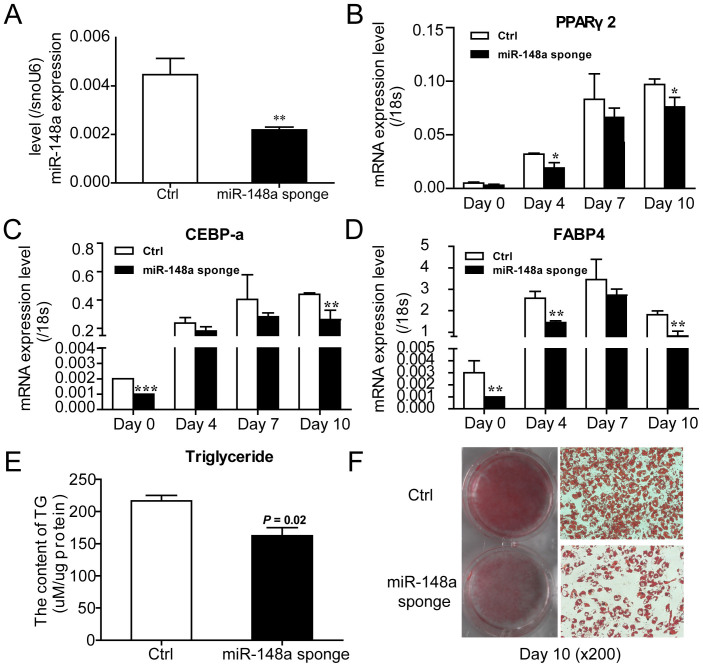
Knockdown miR-148a in hMSCs-Ad inhibits adipogenic differentiation. (A) Knockdown miR-148a efficiency was verified by qRT-PCR at Day 0. (B–D) Knockdown miR-148a in hMSCs-Ad inhibited adipogenic maker gene transcription based on qRT-PCR at Days 0, 4, 7, and 10 during adipogenesis. (E) Triacylglycerol content detected neutral lipid accumulation. (F) Oil red O staining indicated the effect of miR-148a silencing on hMSCs-Ad adipogenic differentiation at Day10. Related mRNA levels are represented as mean ± SEM from three or more independent repeats. *P < 0.05, **P < 0.01, ***P < 0.001.

The effects of miR-148a (miRNA sponge) loss on adipogenesis were also determined. The expression of miR-148a was decreased by about 60% at Day 0 compared with the control (Fig. 3A). Meanwhile, knockdown of miR-148a obviously inhibited adipogenesis, as indicated by the expression of adipocyte-specific factors PPARγ2, C/EBP-α, and FABP4 (Figs. 3B–D), triacylglycerol content (Fig. 3E), and oil red O staining (Fig. 3F).

### miR-148a represses Wnt signaling

Considering these results suggested miR-148a as a positive regulator of adipogenesis, possible mechanisms were explored by reporter gene analysis. The Wnt and TGF-β signaling pathways were identified previously to be associated with adipogenesis in hMSC-Ad^24^,^25^. Reporter assays were first validated in HEK293 cells cotransfected with miR-148a or empty lentiviral vectors and reporter vectors, namely, TOPFlash, FOPFlash^26^, SBE4-TLX-2^27^, and TLX2-luc^28^, as indicators of activation of the Wnt, TGF-β, and BMP pathways (Fig. S3). TOPFlash activity normalized to either Renilla-TK or FOPFlash was inhibited threefold (Fig. S3A). By contrast, TGF-β (Fig. S3B) and BMP signaling (Fig. S3C) were unchanged by miR-148a.

### Identification of miR-148a-binding sequence in target genes

**Figure 4:**
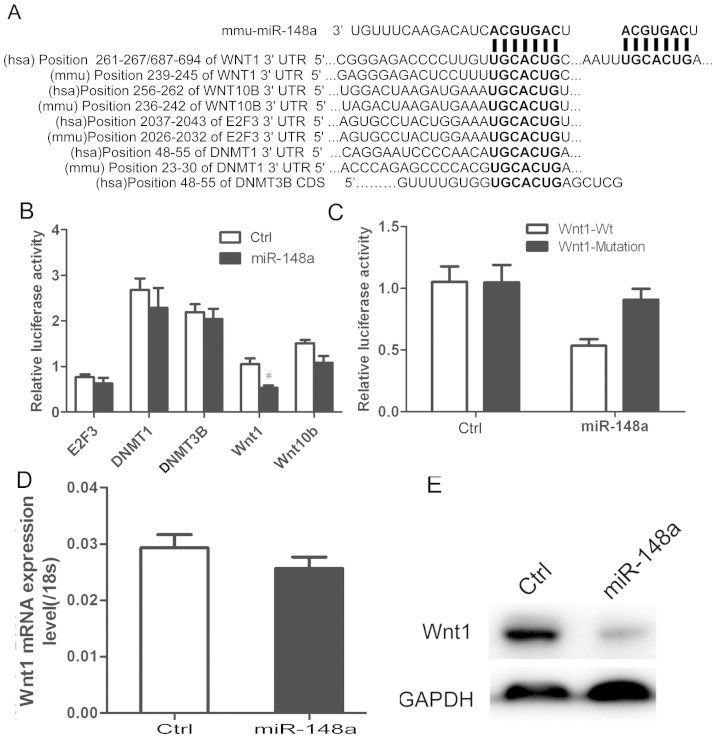
Identification of miR-148a-binding sequence in target genes. (A) Predicted interaction between miR-148a and its putative binding sites in the 3′ UTR or CDS of target gene. Luciferase activity of HEK293 cells cotransfected with reporter vector containing either wild-type (B) or mutant Wnt1 3′-UTR and miR-148a or control (C). (D) qRT-PCR analysis of Wnt in hMSCs-Ad after stable infection with miR-148a or control lentivirus. (E) Western blot analysis of Wnt1 in hMSCs-Ad lysates after stable infection with miR-148a or control lentivirus. GAPDH blot served as loading control. **P < 0.01. Results are mean ± SEM of triplicate measurements (*n* = 4).

To further understand the mechanism underlying the regulation of adipogenesis by miR-148a, candidate target genes were examined by bioinformatics analysis using TargetScan, miRanda, and PicTar. Our results revealed that human and mouse adipocyte differentiation-related molecules, namely, Wnt (Wnt1, Wnt10b), cell cycle (E2F3), and DNA methylation (DNMT1, DNMT3B), were the candidate targets of miR-148a (Figs. S4A and S4B). Analysis revealed potential miR-148a binding sites in the 3′-UTRs of Wnt1, Wnt10b, E2F3, and DNMT1, as well as in the coding sequence of DNMT3B (Fig. 4A). To verify miR-148a binding, the 3′-UTRs of Wnt1, Wnt10b, E2F3, and DNMT1 and the DNMT3B cDNA were cloned into pSi-Check2, then cotransfected with miR-148a or control vector into HEK293 cells. Reporter activity of E2F3, DNMT1, and DNMT3B was unaffected by miR-148a (Fig. 4B). By contrast, miR-148a inhibited activity of Wnt1 but not Wnt10b by 50% (Fig. 4B). Mutation of the miR-148a binding site abolished its inhibitory effect on Wnt1 reporter activity (Fig. 4C). Additionally, miR-148a decreased the expression of Wnt1 protein but not Wnt1 mRNA in hMSCs-Ad (Figs. 4D and 4E), suggesting that miR-148a modulated Wnt1 post-transcriptionally.

### miR-148a regulates adipogenesis in hMSC-Ad via Wnt

**Figure 5:**
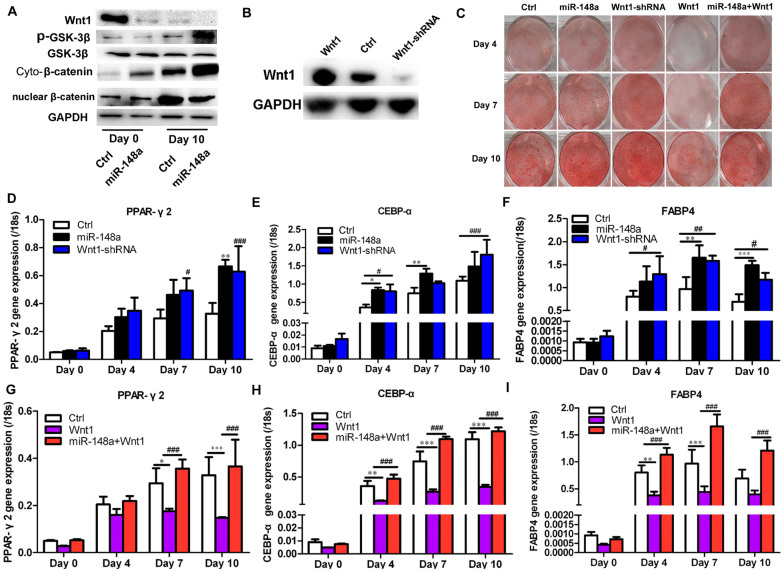
miR-148a regulates adipogenesis in hMSCs-Ad via Wnt. (A) Wnt signal pathway protein level was detected by Western blot. (B) Western blot analysis of Wnt1 in hMSCs-Ad lysates after stable infection with Wnt1-shRNA or Wnt1 construct or control lentivirus. GAPDH blot served as loading control. (C) Oil red O staining indicated the effects of miR-148a on hMSCs-Ad adipogenic differentiation at Days 4, 7, and 10. Transcription levels of adipogenic marker genes, PPARγ2 (D, G), C/EBP-α (E, H), and FABP4 (F, I), were detected by qRT-PCR in different groups at Days 0, 4, 7, and 10 during adipogenesis. *P < 0.05, **P < 0.01, ***P < 0.001; #*P* < 0.05, ##*P* < 0.01, ###*P* < 0.001, compared with Ctrl or Wnt1. Western blot under the same experimental conditions. Data shown are mean ± SEM of four independent experiments. Cyto-β-catenin = cytoplasmic β-catenin; p-GSK-3β = phosphorylation GSK-3β.

Based on the results, the role of Wnt1 in the underlying mechanism of miR-148a-induced adipogenesis was investigated. Canonical Wnt signaling was repressed following miR-148a expression, as indicated by the marked reduction of Wnt1 protein (Fig. 5A, Day 0), increased phosphorylation of GSK-3β (p-GSK-3β) (Fig. 5A, Day 10), and reduced nuclear β-catenin (Fig. 5A, Day 10). Downregulation of endogenous Wnt1 (Fig. 5B) also promoted adipogenesis in hMSCs-Ad (Fig. 5C, Fig. S5) and increased the expression of the adipocyte-related genes PPARγ2 (Fig. 5D), C/EBP-α (Fig. 4E), and Fabp4 (Fig. 5F), consistent with the results of miR-148a overexpression in hMSCs-Ad (Fig. 2). To determine whether miR-148a can rescue Wnt1-suppressed adipogenesis, hMSCs-Ad was co-infected with Wnt1 and miR-148a. Overexpression of miR-148a partially restored differentiation in Wnt1-suppressed hMSCs-Ad as assessed by lipid accumulation (Fig. 5C, last two columns; Fig. S5), as well as PPARγ2 (Fig. 5G), C/EBP-α (Fig. 5H), and Fabp4 (Fig. 4I) mRNA expression.

### Identification of miR-148a promoter

**Figure 6:**
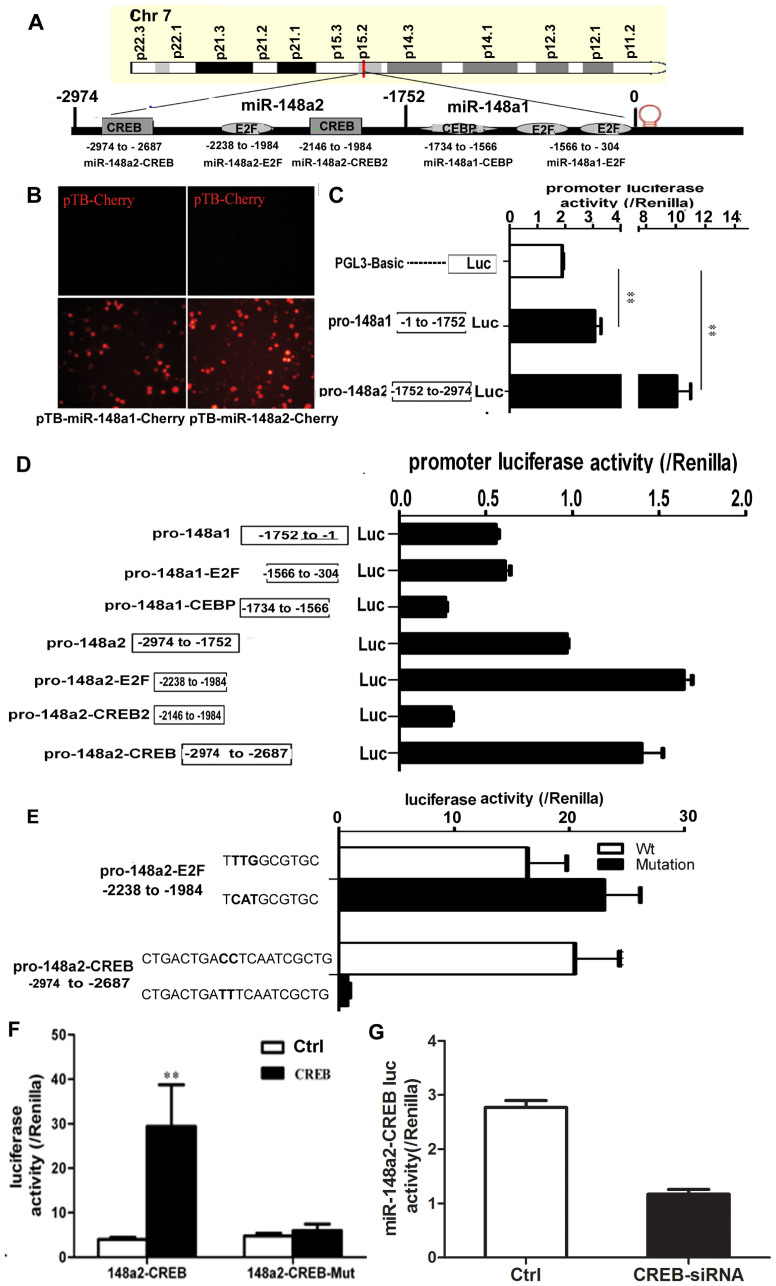
Identification of miR-148a promoter. (A) Schematic of miR-148a promoter and various transcription factor binding sites. (B) HEK293 cells were transfected with pTB-miR-148a1-Cherry (−1 to −1752), pTB-miR-148a2-Cherry (−1752 to −2974), and control pTB-Cherry. Cells were assessed under fluorescence microscope after 48 h post-transfection. Red fluorescence was observed in cells transfected with pTB-miR-148a1-Cherry and pTB-miR-148a2-Cherry. (C) pro-148a1 and pro-148a2 exhibited the highest luciferase activity in comparison with PGL3-Basic. (D) Sequence −2238 to −1984 (pro-148a2-E2F) and −2947 to −2687 (pro-148a2-CREB) were active in HEK293. (E) Mutation of the respective E2F and CREB binding sites in pro-148a2-E2F and pro-148a2-CREB confirmed that pro-148a2-CREB was the major response element. Overexpression (F) and knockdown (G) CREB in HEK293 were used to examine the promoter activity of miR-148a by Luciferase assay. Comparable increase in CREB-dependent activity and mutation of CREB-binding site eliminated transcriptional activity. **P < 0.01, *n* = 4. Data shown are representative of three similar experiments.

The UCSC browser identified a putative promoter region in the miR-148a locus at Chr7p15.2: 25,986,556–25,989,530 (Fig. 6A). The predicted pre-miR-148a promoter region of 2,974 bp was cloned into the pTB-Cherry vector to generate plasmids pTB-miR-148a1-Cherry (−1752 to −1) and pTB-miR-148a2-Cherry (−2974 to −1752), and the activity was detected by fluorescence microscopy upon transfection of HEK293 cells (Fig. 6B). Sequences −2974 to −1752 and −1752 to −1 expressed in a luciferase reporter plasmid indicated that sequence −2974 to −1752 (pro-148a2) was considerably more active than −1752 to −1 (pro-148a1) (Fig. 6C).

To determine the adipogenesis-related transcription factors associated with the miR-148a promoter region, putative response elements were identified using TFSEARCH (http://mbs.cbrc.jp/research/db/TFSEARCH.html). The results revealed putative binding sites for CREB, E2F and CEBP (Fig. 6A). Analysis of the activities of these response elements using a luciferase reporter plasmid revealed that sequence −1566 to −304 (pro-148a1-E2F) and −1734 to −1566 (pro-148a1-CEBP) were equally or less active compared with pro-148-a1, whereas sequence −2238 to −1984 (pro-148a2-E2F) and −2974 to −2687 (pro-148a2-CREB) were more active, and −2146 to −1982 (pro-148a2-CREB2) was less active compared with pro-148a2 (Fig. 6D). Mutation of the corresponding E2F and CREB binding sites in pro-148a2-E2F and pro-148a2-CREB confirmed that pro-148a2-CREB, not pro-148a2-E2F, was the major response element that drives promoter activity (Fig. 6E). Reporter gene activity in HEK293 cells also showed a comparable increase in CREB-dependent activity, and mutation of the CREB binding site eliminated transcriptional activity (Fig. 6F). When the CREB was silenced, transcriptional activity of pro-148a2-CREB was also eliminated (Fig. 6G).

### CREB is bound to miR-148a promoter in hMSC-Ad

**Figure 7:**
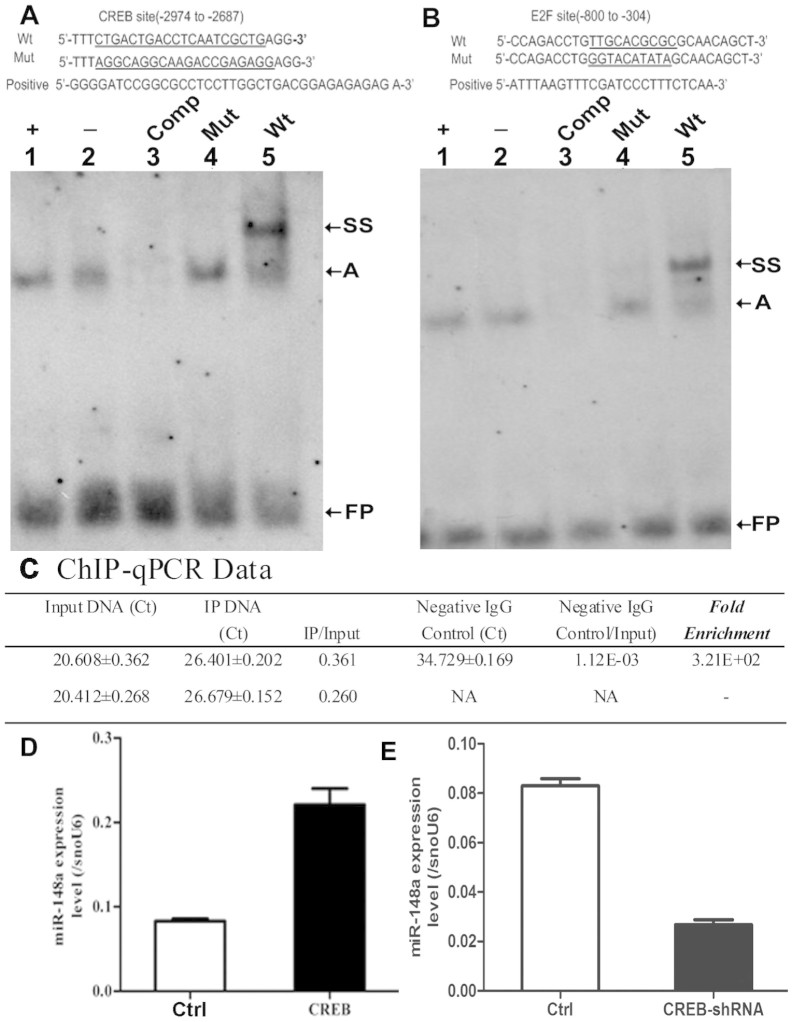
CREB is bound to miR-148a promoter in hMSCs-Ad, and CREB is required for miR-148a expression. EMSA analysis was performed with extracts of hMSCs-Ad with the CREB/E2F binding site. Nuclear proteins extracted from hMSCs-Ad were incubated with digoxigenin-ddUTP-labeled CREB probe. The probe of digoxigenin-ddUTP-labeled CREB/E2F was incubated with cancer cell in lane 1 as positive (+). The probe of digoxigenin-ddUTP-labeled CREB/E2F was incubated with Nuclear proteins extracted from hMSCs-Ad in lane 2 were absent (−). 50-fold excess of unlabeled cold competitors (Comp; lane3), 50-fold excess of unlabeled mutated CREB/E2F (Mut; lane 4), the probe of digoxigenin-ddUTP-labeled wild-type CREB/E2F (Wt; lane5). (A) EMSA with 50-fold cold competitors of Wt, mutant oligonucleotides (Mut). Sequences of double-stranded DNA probe containing the predicted Wt CREB (nt −2974 to nt −2687) binding domain, positive, and mutation oligonucleotides (Mut) used in EMSA analysis. EMSA analysis was performed with the E2F binding site. CREB bound to the promoter region of miR-148a in hMSCs-Ad (lane 5). (B) Sequence of double-stranded DNA probe containing the predicted E2F binding site (nt −800 to nt −304), positive, and mutation oligonucleotides (Mut). Supershift band was observed in lane 5. (C) ChIP-qPCR was performed with extracts of hMSC-Ad with the CREB/E2F binding site. ChIP-qPCR assay showing the fold enrichment of CREB binding to miR-148a promoter. Results were analyzed using ΔΔCt method. ChIP Ct values were normalized for background by using the Ct value for a mock IP fraction. Overexpression (D) and knockdown (E) CREB in hMSC-Ad were used to examine the expression level of miR-148a by qRT-PCR. miR-148a was highly expressed in CREB overexpressed hMSC-Ad (*n* = 3). **P < 0.01. *n* = 4. Data shown are representative of three similar experiments. The major complex of nuclear extract-bound probe, designated A, are indicated with arrows to the right of the gels. + = positive; − = absent; FP = free probe; SS = super-shift; Comp = competitor.

To further characterize the essential regulatory elements of the miR-148a promoter for subsequent genetic analysis, electrophoretic mobility shift analysis (EMSA) was used to verify the transcription factors bound to this region. Lanes 1 and 2 confirmed the quality of probe and sample (Figs. 7A and 7B). EMSA revealed that CREB and E2F bound to promoter regions −2974 to −2687 and −800 to −304, respectively (Figs. 7A and 7B; “Mut” and “Wt,” lanes 4 and 5). Binding was supershifted with an anti-CREB or anti-E2F antibody (Figs. 7A and 7B; “Wt,” lane 5) and can compete with unlabeled DNA containing the wild type but not the mutated sequence (Figs. 7A and 7B; “Comp,” lane 3). In comparison, sequences −2146 to −1984 (CREB), −1734 to −1566 (CEBP), and −1566 to −800 (E2F) did not bind to the miR-148a promoter region (Fig. S6). To further examine the interaction of CREB and E2F with the miR-148a promoter region in vivo, ChIP analysis was performed in hMSCs-Ad (Fig. 7C). In this procedure, chromatin is isolated and subjected to cross-linking and shearing of the DNA prior to IP with antibodies against specific proteins. The association of the protein of interest with the miR-148a promoter was assessed by real-time PCR using primers specific for the miR-148a promoter after the reversal of cross-linking. The IP of CREB was enriched 320-fold compared with input DNA and the same CREB, but not E2F, bound to the miR-148a promoter sequence.

Therefore, we examined whether endogenous miR-148a expression was regulated by CREB in hMSCs-Ad. Following transduction of hMSCs-Ad with a lentivirus expressing CREB, the level of miR-148a increased 2.6-fold compared with control cells (Fig. 7D). By contrast, the level of miR-148a decreased by about 70% upon CREB knockdown (Fig. 7E).

### Differential miR-148a expression in obese mice and human subjects

**Figure 8:**
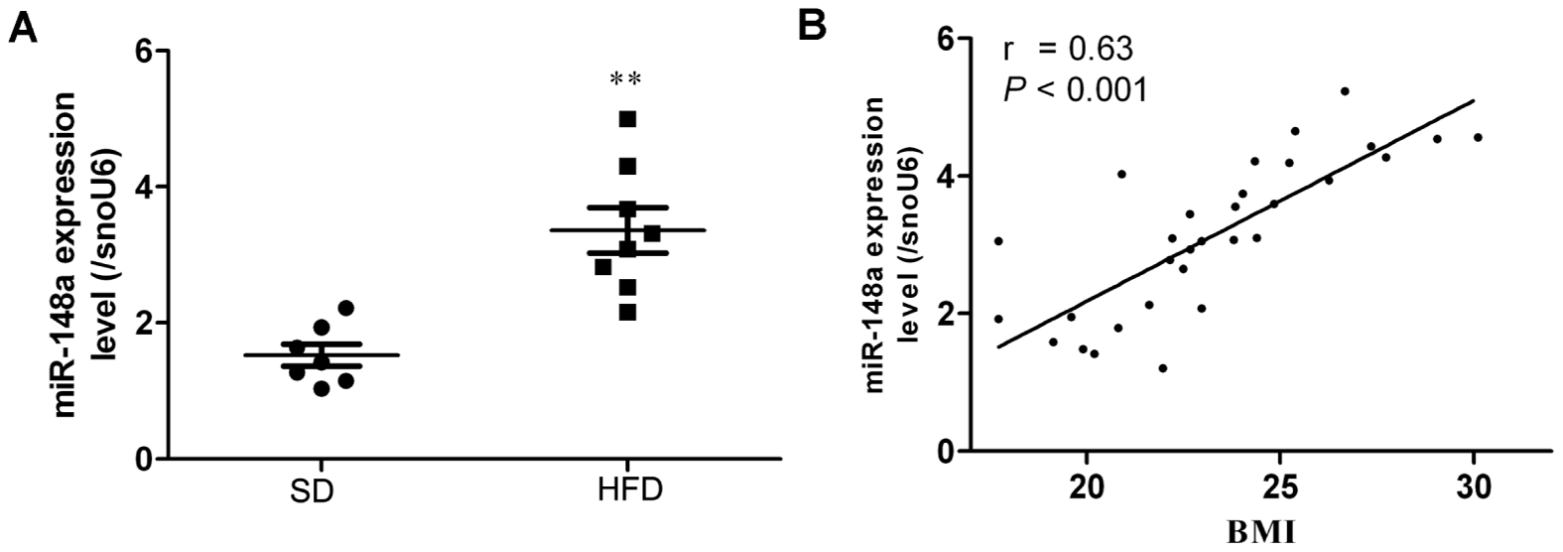
Differential miR-148a expression in lean and obese human subjects and mice. (A) miR-148a expression levels in epididymal fat pads of SD or HFD mice were determined by qRT-PCR. (B) Positive correlation existed between miR-148a levels and BMI. **P < 0.01. Data shown are representative of three similar experiments.

To assess the utility of miR-148a as a biomarker in the development of obesity, miR-148a expression was measured in adipose tissues from obese eight-week-old C57BL/6J mice fed high-fat diet (HFD) and compared with age-matched controls fed standard diet (SD). Mice fed HFD for 17 weeks exhibited an increase in body weight and hyperglycemia compared with littermate controls fed SD (Figs. S7A–S7C). Epididymal adipose tissues from mice fed HFD significantly increased in miR-148a expression compared with animals fed SD (Fig. 8A). By contrast, brown adipose tissues from mice fed HFD decreased in miR-148a expression compared with animals fed SD (Fig. S7D). Abdominal fat biopsies were also obtained from patients undergoing surgery for abdominal disorders (Supplementary Table 1). Expression of miR-148a increased in these subjects in proportion to increasing BMI (Fig. 7B, *r* = 0.63, *P* < 0.001, Pearson’s correlation). Thus, these data in both mouse model and human subjects suggested a correlation between miR-148a and development of obesity.

## Discussion

hMSCs-Ad can differentiate into several lineages, including adipocytes, in response to stimulation by multiple environmental factors^29^. To achieve better understanding of the roles of miRNAs in adipogenesis, expression of miRNAs was analyzed. The levels of miR-148a, miR-26b, miR-30, and miR-199a were increased in differentiating hMSCs-Ad. Among these miRNAs, miR-148a exhibited the most effects on increasing PPRE luciferase activity. Thus, the role of miR-148a in obesity was clarified in the present study. miR-148a was highly expressed in hMSCs-Ad during adipogenesis relative to its negative regulatory effect on Wnt1 expression. This new role for miR-148a was only previously recognized as modulator function of tumor suppressor genes in gastrointestinal tumors^30^. However, previous studies on hMSCs-Ad undergoing adipogenesis reported that miR-21^13^, miR-22^14^, miR-196^15^, miR-27b^20^, and miR-138^31^ were either upregulated or downregulated, and miR-148a was not reported in hMSCs-Ad. Thus, a relatively little overlap existed between these results and our current findings. Our data also showed a 10-fold increase in miR-148a in differentiated 3T3-L1 preadipocytes in accordance with the study of Xie et al.^16^, which supported our thesis that miR-148a may play a key role in adipogenesis. This finding is also supported by the high expression of miR-148a in diet-induced obese mice, as well as our finding that miR-148a levels in human adipose tissues achieve proportion to an increase in BMI, providing new insights into the roles of miRNAs in obesity and related metabolic disorders.

Exogenous expression of miR-148a in hMSCs-Ad significantly increased TG content, GPDH activity, and adipogenesis relative to an increase in differentiation-specific factors at both transcriptional and translational levels. This effect was partially attributable to the upregulation of PPAR-dependent transcription, a major factor in adipogenesis^23^, and to inhibition of Super TOPFlash activity, a Wnt-depend transcription. Qin et al.^32^ found that miR-148a was downregulated in activation of Wnt signaling in 3T3-L1 cells. As mentioned above, whether miR-148a acted as an additional adipogenic regulator in hMSCs-ad or differentiation factor should be elucidated.

This hypothesis was first proved by identification of a direct miR-148a target protein. Bioinformatics analysis using TargetScan, miRanda, and PicTar revealed that the Wnt (Wnt1, Wnt10b), cell cycle (E2F3), and DNA methylation pathways (DNMT1, DNMT3B) were candidate targets of miR-148a. The TGF-β signal pathway has been described as anti-adipogenic^24^. Bone morphogenetic proteins (BMPs) belong to the TGF-beta superfamily, and BMP4 plays a critical role in adipogenesis^33^. In our study, we examined the effect of miR-148a on TLX-2 reporter gene, which is a downstream target for BMP signaling in the primitive streak where BMP-4 and other TGF-related factors are expressed. Our results showed that TLX-2 luciferase activity was unchanged by miR-148a. Fajas et al^34^ found that 3T3-L1 cells are arrested in the G1 phase of the cell cycle after the initial phase of DNA replication, inducing gene expression for fat accumulation. Pinnick et al.^35^ have reported that the contribution of DNA methylation to the determination of cells of adipogenic fate is very critical. Our results showed that miR-148a cannot bind to the 3′-UTRs of E2F and DNMT1/3B.

Interestingly, miR-148a can inhibit the Wnt1 expression at post-transcriptional level. We further identified Wnt1 as a direct translational target of miR-148a, where it directly bound to the 3′-UTR of Wnt1 and not to the related Wnt10b, which is also consistent with previous studies^36^. A recent study demonstrated that miR-148a silencing resulted in Wnt10b-mediated stimulation of tumor cell motility in cancer-associated fibroblasts^36^,^37^. However, other studies have found little or no change in Wnt1 or Wnt10b expression by miR-148a or miR-148b in gastric cancer cells^38^,^39^, suggesting that inhibition is dependent on cellular context. Knockdown of Wnt1 by RNA interference blocked adipogenesis of hMSCs. Wnt1 or Wnt10b inhibits the differentiation of mesenchymal stem cells^40^ and blocks adipogenesis in vivo^41^; thus, miR-148a expression rescued the negative effect of Wnt1 expression in hMSCs-Ad. These findings suggest that miR-148a is an upstream regulator rather than an effector of adipogenesis. Knockdown of miR-148a also significantly influenced adipogenesis compared with the control.

Increasing evidence reported that miRNAs are processed from long primary transcripts (pri-miRNAs), which are transcribed by RNA polymerase II and subjected to regulation by a multitude of transcription factors^42^,^43^,^44^. Promoter analysis of the miR-148a locus indicated it as an intergenic miRNA with putative CREB binding sites in the core promoter region. EMSA and ChIP analyses demonstrated that CREB bound to the miR-148a promoter in hMSCs-Ad, suggesting interplay between CREB and the pri-miR-148a proximal promoter. CREB can trigger adipogenesis by binding to regulatory elements in the promoters of adipocyte-specific genes, including PEPCK, FABP, FAS, and CEBP-β in a coordinated manner with other regulatory factors^45^. CREB overexpression in 3T3-L1 preadipocytes promoted the expression of adipocyte markers and the accumulation of triglycerides^7^. Adipogenesis is also tightly regulated by several transcription factors, including PPARγ and CREB^6^,^7^. Our findings showed that CREB increased miR-148a levels and its promoter activity in hMSCs-Ad, indicating its critical involvement in modulating miR-148a expression during adipogenesis.

The presence of brown fat has been demonstrated in adult humans^46^, making this tissue a potential target for the treatment of obesity and metabolic syndromes^47^. We tested whether miR-148a levels were altered in brown adipose tissues and observed downregulated levels, opposite to the changes observed in epididymal white adipose tissues. These findings suggested that miR-148a may function differently in brown fat tissues to influence the occurrence and development of mouse obesity.

In summary, our data provide the first evidence of miR-148a as a CREB- dependent and adipogenic-specific miRNA in hMSCs-Ad, which mediates this effect through modulation of Wnt signaling. Importantly, miR-148a as a biomarker of obesity was elevated in adipose tissues of obese mice, as well as in human subjects. Thus, our findings provide a basis for the role of miR-148a in adipogenesis, which may underlie the development of obesity and associated metabolic disorders. Further research may elucidate whether miR-148a can modulate adipogenesis in vivo and possibly provide a novel therapeutic target for the management of obesity.

## Methods

### Animals and diets

Animal protocols were approved in accordance with the guidelines established by the Research Animal Care Committee of Nanjing Medical University. Eight-week-old male C57BL/6J mice (Jackson Laboratory, Bar Harbor, ME) were individually caged and distributed into weight-matched groups then fed either SD (4.5% fat) or HFD (60% fat; Research Diets, New Brunswick, NJ). Body weight was determined weekly after initiating HFD.

### Human subjects

Abdominal fat biopsies were prospectively collected from patients undergoing surgery for abdominal disorders. None of the patients had any type of endocrine disorder, malignancy, or severe systemic illness as determined by procedures approved by the Ethics Committee of the Nanjing Maternity and Child Health Care Hospital Affiliated to Nanjing Medical University, PRC (Nos. 2011-50 and 2013-4). Subjects with BMI ≥ 24 kg/m^2^ were considered overweight according to the Working Group on Obesity in China (WGOC) in 2003^48^. All human participants provided written informed consent. The methods were carried out in accordance with the approved guidelines of Ethics Committee of the Nanjing Maternity and Child Health Care Hospital Affiliated to Nanjing Medical University, PRC. The experimental protocols were approved by the Ethics Committee of the Nanjing Maternity and Child Health Care Hospital Affiliated to Nanjing Medical University, PRC (Nos. 2011-50 and 2013-4).

### Cell culture and adipocyte differentiation

hMSCs-Ad (Cat. No. 7510, ScienCell Research Laboratories, San Diego, CA) were characterized by flow cytometry with surface markers of antibodies (Fig. S8). Thus, these cells were of more pure population of stem cells and slightly different with stromal vascular fractions, defined as “primary” preadipocytes. hMSCs-Ad were maintained in Mesenchymal Stem Cell Medium (MSCM) (Cat. No. 7501, ScienCell Research Laboratories) supplemented with 5% fetal bovine serum, 1% mesenchymal stem cell growth supplement (Cat. No. 7501), and 1% penicillin/streptomycin solution at 37°C in a humidified atmosphere under 5% CO_2_. To induce differentiation, hMSCs-Ad were incubated in serum-free MSCM supplemented with 50 nM insulin, 100 nM dexamethasone, 0.5 mM 3-isobutyl-1-methylxanthine, and 100 μM rosiglitazone (Day 0). The medium was replaced every 2 d for 4 d. Cells were then incubated in serum-free MSCM supplemented with 50 nM insulin and replaced every 2 d until lipid accumulated in cells (Day 10). hMSCs-Ad were harvested on Days 0, 4, 7, and 10 and washed twice with l ml of PBS at 4°C. Neutral lipid accumulation was measured by staining formalin-fixed cells with Oil Red O. Adipogenic-specific glycerol-3-phosphate dehydrogenase (GPDH) enzymatic activity was measured in triplicate wells by using the method described previously^49^. Intracellular triglyceride (TG) content was measured using a triglyceride assay kit (GPO-POD, Applygen Technologies Inc., Beijing, China) in accordance with the manufacturer’s protocol. Protein concentration was measured using BCA Protein Assay Kit (Thermo Fisher Scientific, Waltham, MA). Results are expressed as µmoles TG/mg protein. Expression of the adipocyte markers PPARγ2, Fabp4, and C/EBP-α were measured by qRT-PCR as described below.

### Plasmids and reporter assays

The miR-148a lentiviral expression vector pGLV3-H1-GFP-puro-miR-148a, miR-148a sponge (four repeat complimentary sequences) lentiviral expression vector, and the negative control vector pGLV3-H1-GFP-puro were purchased from GenePharma (Shanghai, China). The miR-148a minigenes, including the upstream and downstream sequences of the pre-miRNA and open reading frame of CREB and Wnt1, were amplified by PCR and ligated into the *Bam*HI and *Eco*RI sites of pGLV3-H1-GFP-puro. Human embryonic kidney 293T cells (HEK293T, American Type Culture Collection, Manassas, VA) were grown in DMEM supplemented with 10% FBS, 4 mM L-glutamine, and 100 µg/ml Normocin. HEK-293T cells were transfected with lentiviral packaging vectors (ABM, Richmond, BC, CA) and lentiviral vectors expressing miR-148a, CREB, or Wnt1 by Lipofectamine 2000. Viruses were collected after 48 h. Infection efficiency was monitored by GFP expression using fluorescence microscopy. For overexpression studies, subconfluent hMSCs-Ad were infected. After the cells reached confluency, they were transferred to adipocyte-differentiation medium. Adipocyte differentiation was induced, and the cells were harvested at various time points for protein and mRNA assays.

Partial sequences from the 3′ UTRs of Wnt1 (nt 261 to 267 and nt 687 to 693), Wnt10b (nt 255 to 261), E2F3 (nt 2037 to 2043), DNMT1 (nt 48 to 55), and CDS of DNMT3B (nt 431 to 437) were amplified by PCR and cloned into the *Xho*I and *Not*I sites of pSi-CHECK-2 (Promega, Madison, WI). The miR-148a-binding sites in the reporter vector were mutated using the Quick Change Site-Directed Mutagenesis Kit (Agilent Technologies Inc., Santa Clara, CA) in accordance with the manufacturer’s instructions. All constructs were verified by sequencing. HEK293 cells (10,000 per well) were grown in DMEM supplemented with 10% FBS, 4 mM L-glutamine, and 100 µg/ml Normocin. The cells were seeded into 96-well plates. After 24 h, the cells were transfected with 50 ng of pSi-CHECK-2 and 100 ng of pGLV3-H1-GFP-puro-miR-148a or the control virus using 0.3 μl of Lipofectamine 2000 (Invitrogen, Life Technologies Corporation, Carlsbad, CA). After 48 h, Renilla and Firefly luciferase activities were assayed with the Dual-Glo® Luciferase Assay System (Promega, Madison, WI). Assays were performed in triplicate and repeated thrice.

### Bioinformatics and identification of miR-148a target sites

TargetScan (http://www.targetscan.org)^50^, PicTar (http://pictar.mdc-berlin.de)^51^, and miRanda (http://www.microrna.org) were used to predict the target consensus sequences for miR-148a. The 3′-UTR (3′-untranslated region) sequences were searched for perfect antisense matches to the designated seed region of each miRNA (bases 2–8 from the 5′ end). Significantly overrepresented miRNA motifs in the 3′-UTRs of genes in each cluster and for the full set of differentially regulated genes that were significantly overrepresented compared with the motifs in the whole 3′-UTR sequence set were determined using one-sided Fisher’s exact test (*P* < 0.05). To account for multiple testing, *P*-values were adjusted by controlling for false discovery rate (< 5%)^52^. Results were analyzed by Gene Ontology (GO) and pathway analysis.

### Multi-pathway reporter-scan assays

A total of 10^4^ HEK293 cells were grown in DMEM supplemented with 10% FBS, 4 mM L-glutamine, and 100 µg/ml Normocin. These cells were seeded into 96-well plates. After 24 h, the cells were transfected with 100 ng of pGLV3-H1-GFP-puro-miR-148a or control vector and 100 ng of the reporter genes PPRE × 3-TK (Addgene, Cambridge, MA)^23^, Super 8x TOPFlash/Super 8x FOPFlash (Super 8x TOPFlash mutant) (Addgene, Cambridge, MA)^26^, SBE4 (Addgene, Cambridge, MA)^27^, and TLX-2 (Addgene, Cambridge, MA)^28^, as well as 2.5 ng of Renilla luciferase vector (pRL-TK), using Lipofectamine 2000 in accordance with the manufacturer’s instructions. After 48 h, the cells were lysed in Passive Lysis Buffer and 20 μl of the sample was used to measure luciferase activity using Dual-Luciferase Assay (Promega, Madison, WI). Activity was expressed as Firefly luciferase activity normalized to Renilla luciferase activity. Each assay was performed in triplicate and repeated thrice.

### Promoter analysis

The upstream region of miR-148a was examined using the UCSC genome browser (http://genome.ucsc.edu/). The predicted response elements were determined with TFSEARCH (http://diyhpl.us/~bryan/irc/protocol-online/protocol-cache/TFSEARCH.html). The minimum match is the conservation reached in at least 80% of sites, and the minimum match number of sites is 5. The predicted promoter regions, namely, pTB-miR-148a1-Cherry (−1 to −1752) and pTB-miR-148a2-Cherry (−1752 to −2974) of miR-148a (chr7p15.2: 25,986,556–25,989,530, 2974 bp), were cloned into pTB-Cherry constructed from pTA-Luc (Clontech, Mountain View, CA) and pTB-Cherry^53^ using the *Nsi*I and *Xho*I restriction sites. The promoter region was divided into seven sequences (Fig. 5A), amplified by PCR, and subcloned into pGL3-Basic (Promega, Madison, WI). All sequences were confirmed by sequencing. Primers are shown in Supplementary Table 2.

### Fluorescence and promoter activity

HEK293 cells were grown to 60% to 70% confluence in six-well plates and transfected with 2 μg reporter plasmids per well using Lipofectamine 2000. At 48 h post-transfection, fluorescence was observed by fluorescence microscopy. Promoter activity was measured by transfecting cells with 250 ng/well of promoter–Firefly luciferase reporter construct and 25 ng/well of Renilla luciferase vector (pRL-TK) using 0.6 μl of Lipofectamine 2000 in 20 μl of Opti-MEM® I Reduced-Serum Medium (Life Technologies, Grand Island, NY). After 24 h, cells were lysed in 50 μl of 1× Passive Lysis Buffer and stored at −20°C until assayed. Luciferase activity was measured using the Dual-Luciferase Reporter Assay System (Promega, Madison, WI) as described above.

### RNA isolation and quantitative RT-PCR

Total RNA was prepared from hMSC-Ad or 3T3-L1 cells at varying intervals after induction of adipocyte differentiation by using TRIzol (Invitrogen, Carlsbad, CA) in accordance with the manufacturer’s protocol, followed by DNase treatment (TaKaRa, Japan). Quality and concentration of RNA was assessed by NanoDrop 2.0 (Thermo Fisher Scientific, Waltham, MA). cDNA was synthesized from 200 ng of RNA using TaqMan miRNA Reverse Transcriptase Kit (ABI, Foster City, CA). Adipocyte-differentiation markers PPARγ2, C/EBP-α, and FABP4 were measured using 480 ng of RNA and High-Capacity cDNA Reverse Transcription Kit (ABI, Foster City, CA). RT-PCR was performed using the Applied Biosystems 7500 Sequence Detection System (ABI 7500 SDS; Foster City, CA) in accordance with the manufacturer’s guidelines. Briefly, samples were incubated at 95°C for 10 min for an initial denaturation, followed by 40 PCR cycles consisting of incubation at 95°C for 15 s and 60°C for 1 min; miRNA expression was normalized to snoU6. The primer and probe sequences for the marker genes are presented in Supplementary Table 2. Relative gene expression levels of miRNA or mRNA were quantified based on the cycle threshold (Ct) values and normalized to snoU6 and 18S ribosomal RNA, respectively. Each sample was measured in triplicate, and gene expression levels were calculated by 2^−ΔΔct^ method.

### Western blot

hMSCs-Ad were lysed in immunoprecipitation (IP) assay buffer (150 mM NaCl, 1.0% IGEPAL CA-630, 0.5% sodium deoxycholate, 0.1% SDS, and 50 mM Tris, pH 8.0) containing a protease inhibitor mixture (Roche Applied Science, Penzberg, Upper Bavaria, Germany). Protein concentrations were determined by the BCA protein assay kit (Thermo Fisher Scientific, Waltham, MA). Proteins were separated on a 10% SDS/PAGE gel under reduction conditions and electroblotted onto a PVDF membrane. Membranes were probed with primary antibodies against PPARγ2 (Cat. No. 2435S, Cell Signaling Technology, Danvers, MA) and C/EBP-α (Cat. No. 8178S, Cell Signaling Technology, Danvers, MA), Wnt1 (Cat. No. 365800 Invitrogen, Carlsbad, CA), β-catenin (Cat. No. 365800, Invitrogen, Carlsbad, CA), phosphorylated GSK-3β (Cat. No. 5558, Cell Signaling Technology, Danvers, MA), and GSK-3β (Cat. No. 9832S, Cell Signaling Technology, Danvers, MA). Membranes were reprobed with anti-GAPDH antibody (Sigma, St. Louis, MO) to normalize expression.

### EMSA analysis

Cells were collected by centrifugation, and nuclear proteins were extracted using the Cellular and Nuclear Protein Extraction Kit (Pierce, Italy). Nuclear protein (10 μg) was used for EMSA to detect CREB and E2F binding activity using a digoxigenin-ddUTP-labeled double-stranded oligonucleotide probe. The sequences of CREB and E2F probes, mutated probes, and binding site consensus sequences (positive probe) are shown in Fig. 7. CREB- and E2F-binding activities were detected using the EMSA Kit (Roche Applied Sciences, Penzberg, Upper Bavaria, Germany) in accordance with the manufacturer’s protocol. For supershift assays, anti-CREB (Cat. No. 17600, Millipore, Billerica, MA) and anti-E2F antibodies (Cat. No.1710061, Millipore, Billerica, MA) were pre-incubated with the labeled probes and bands visualized with the Bio-Rad Gel Imaging System (Bio-Rad, Hercules, CA). For competition studies, a 100-fold excess of the specific unlabeled oligonucleotide probe was added to the binding mixture, and the reaction continued for 10 min before addition of the labeled probe.

### Quantitative chromatin IP

Up to 10^7^ hMSCs-Ad were washed with PBS and cross-linked with 1% formaldehyde for 10 min. Chromatin was sonicated (Sonic Dismembrator, Model 100, Thermo Fisher Scientific, Waltham, MA) on ice 12 times for 10 s each at the highest setting to generate chromatin fragments of 500 bp to 2000 bp. Sonicated chromatin was quantified on the basis of DNA content at A260. Each IP contained 40 μg of DNA chromatin based on its absorption at A260. ChIP analysis was performed with antibodies against CREB, E2F1 (Millipore, Billerica, MA), and the same IgG isotype as control. Input control DNA or immunoprecipitated DNA was amplified in a 15 μl reaction volume containing 2 μl of the eluted DNA template. Four sets of primers were designed to target different regions of the pri-miR-148a promoter (Supplementary Table 3). Both the immunoprecipitated fragments and the inputs were amplified by real-time PCR. The results for the immunoprecipitated fragments were calculated and compared with the input samples in each case, then expressed as a percentage of the input.

### Statistical analysis

All results were obtained from at least three independent experiments and presented as mean ± SEM. Differences between groups were analyzed by Student’s two-tailed *t*-test when only two groups were present or by one-way analysis of variance when more than two groups were compared. Differences with *P*-values < 0.05 were considered significant.

## Author Contributions

C.M.S. researched data and participated in writing of the manuscript. M.Z., L.Y., L.X.P., L.C., Q.H. and G.F.X. collected the clinical samples. C.M.S., M.L.T., C.X. and M.Z. contributed to the discussion. X.R.G. and C.B.J. provided oversight for the project and participated in editing of the manuscript. Y.H.N. contributed to the loss of function experiment partly and participated in editing the revised version. X.R.G. is the guarantor of this work and, as such, had full access to all the data in the study and takes responsibility for the integrity of the data and accuracy of analyses.

## Additional information

**Supplementary information** accompanies this paper at http://www.nature.com/scientificreports

**How to cite this article:** Shi, C. *et al*. miR-148a is associated with obesity and modulates adipocyte differentiation of mesenchymal stem cells through Wnt signaling. *Sci. Rep*. 5, 9930; DOI:10.1038/srep09930 (2015)

## Supplementary Material

Supplementary Information Supplementary Information
