# Supplementary material for: miR-148a is Associated with Obesity and Modulates Adipocyte Differentiation of Mesenchymal Stem Cells through Wnt Signaling
Source: Sci Rep. 2015 May 22;5:9930. doi: 10.1038/srep09930 (PMC4441322; doi:10.1038/srep09930)
Supplement: Supplementary Information [file srep09930-s1.pdf]

miR-148a is associated with Obesity And Modulates Adipocyte Differentiation of Mesenchymal Stem Cells Through Wnt Signaling

Chunmei Shi, Min Zhang, Meiling Tong, Lei Yang, Lingxia Pang, Ling Chen, Guangfeng Xu, Xia Chi, Qin Hong, Yuhui Ni, Chenbo Ji, Xirong Guo

Supplementalry Figure legends

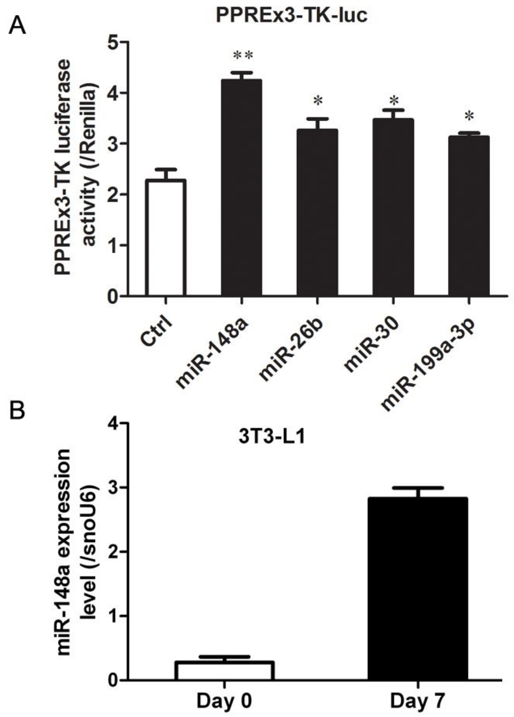

Fig.S1. miR-148a increased PPREx3-TK activity and its expression level during adipogenesis in 3T3-L1 cells. (A) PPREx3-TK activity was more robust when coexpressed with miR-148a than other miRNAs. (B) 3T3-L1 preadipocytes were grown to confluence, and adipogenic differentiation was initiated, as described in Research Design and Methods. The expression of miR-148a was quantified by TaqMan microRNA-based qRT-PCR in 3T3-L1 cells. Data shown are the mean ± SEM of four independent experiments.

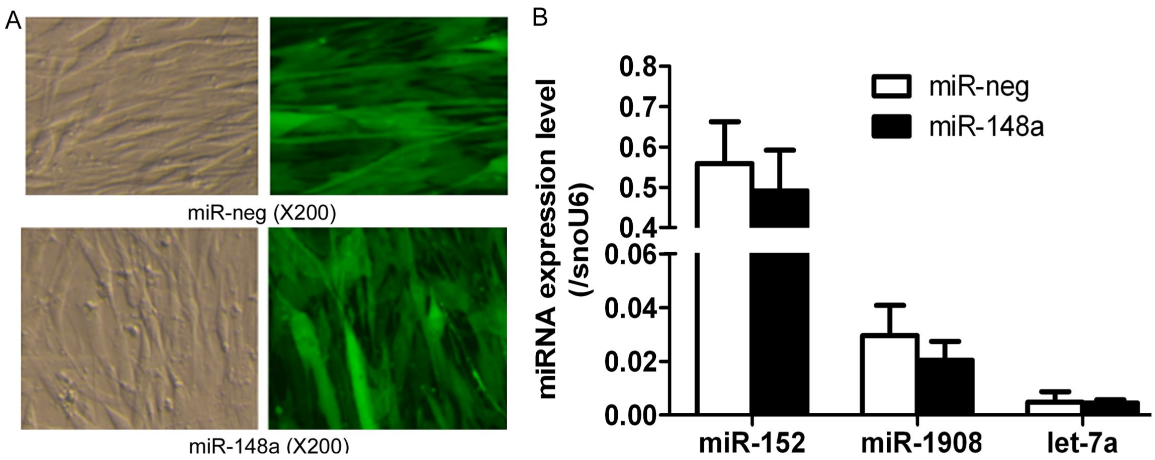

Fig.S2. Overexpression of miR-148a did not interrupt other endogenous miRNA pathways. (A) hMSCs-ad were infected with lentivirus of miR-148a or lentivirus alone control for 48 h before differentiation inducer, 95% of cells were GFP positive as measured by fluorescent microscopy. (B) miR-152, miR-1908 and let-7 expression levels were detected by qRT-PCR. Data shown are the averages of three independent experiments.

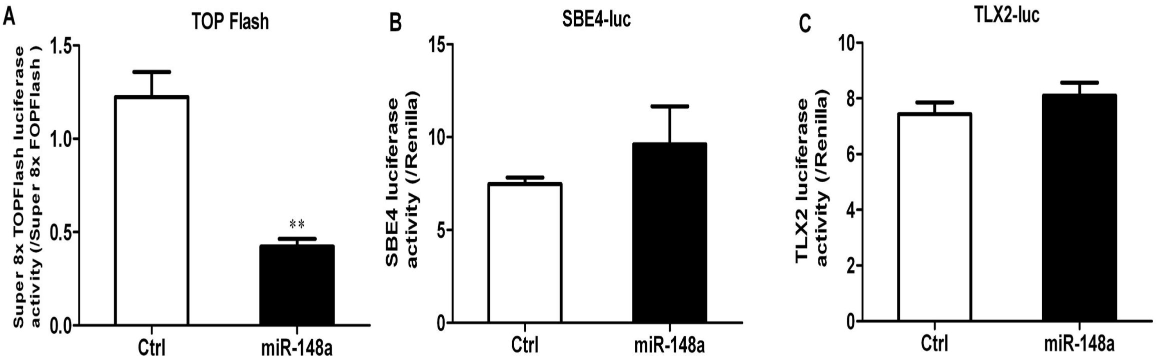

Fig.S3. miR-148a represses Wnt signaling by multiple pathway scan. miR-148a regulated the pathway, which was determined by multi-pathway reporters-scan assays in HEK293 cells (n=4). (A) miR-148a suppressed Super TOPFlash reporter activity. (B, C) TGF-β and BMP signaling were unchanged by miR-148a expression. HEK293 cells were cotransfected with the miR-148a or control lentivirus vector and reporter vectors. Luciferase activity was measured after 24 h. Results are mean ± SEM of triplicate measurements.

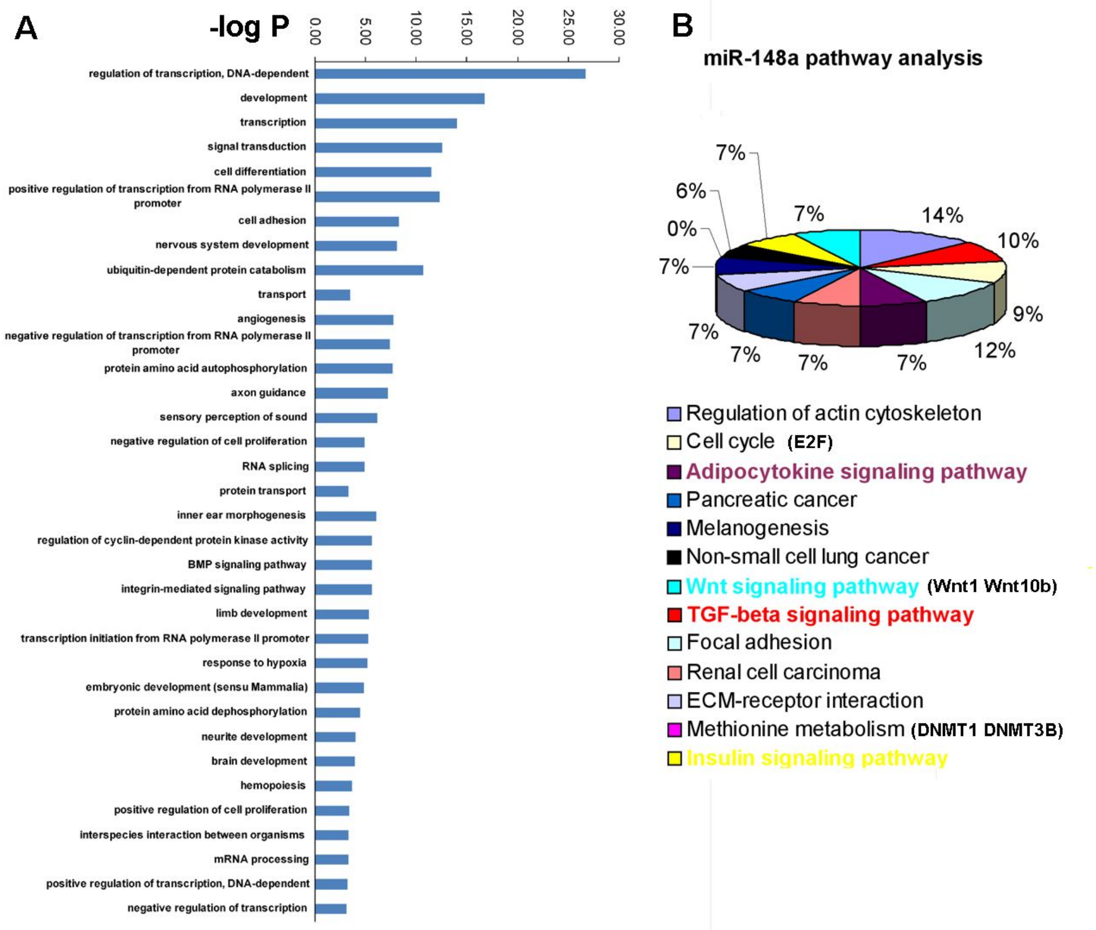

Fig.S4. Bioinformatics analysis target genes of miR-148a. (A) DAVID Bioinformatics Resources 6.7 was used to perform statistical analysis of over represented GO terms to predict target genes of miR-148a. Cellular processes were sorted by score (-log [*P* value]). Highly positive score set included genes involved in the Wnt signaling, cell cycle and DNA methylation pathwayS. (B) The pathway analyses for predicting target genes of miR-148a.

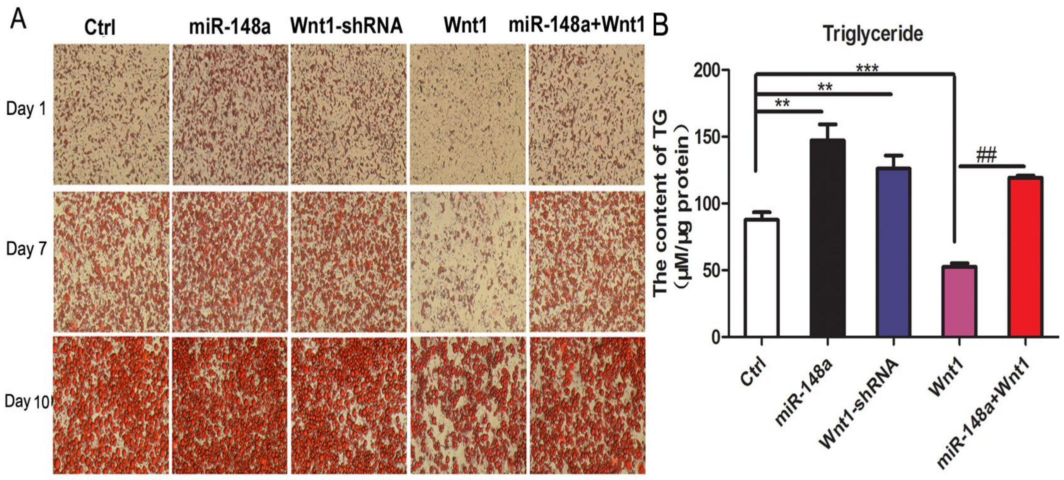

Fig.S5. Overexpression of miR-148a partially restored differentiation in Wnt1-suppressed hMSCs-Ad. (A) Oil red O staining indicated the effect of miR-148a overexpression on hMSCs-Ad adipogenic differentiation at Days 1, 7 and 10. (B) Triacylglycerol content detected neutral lipid accumulation. Data shown are representative of three similar experiments. ***P* <0.01, ****P* <0.001, ##*P* <0.01.

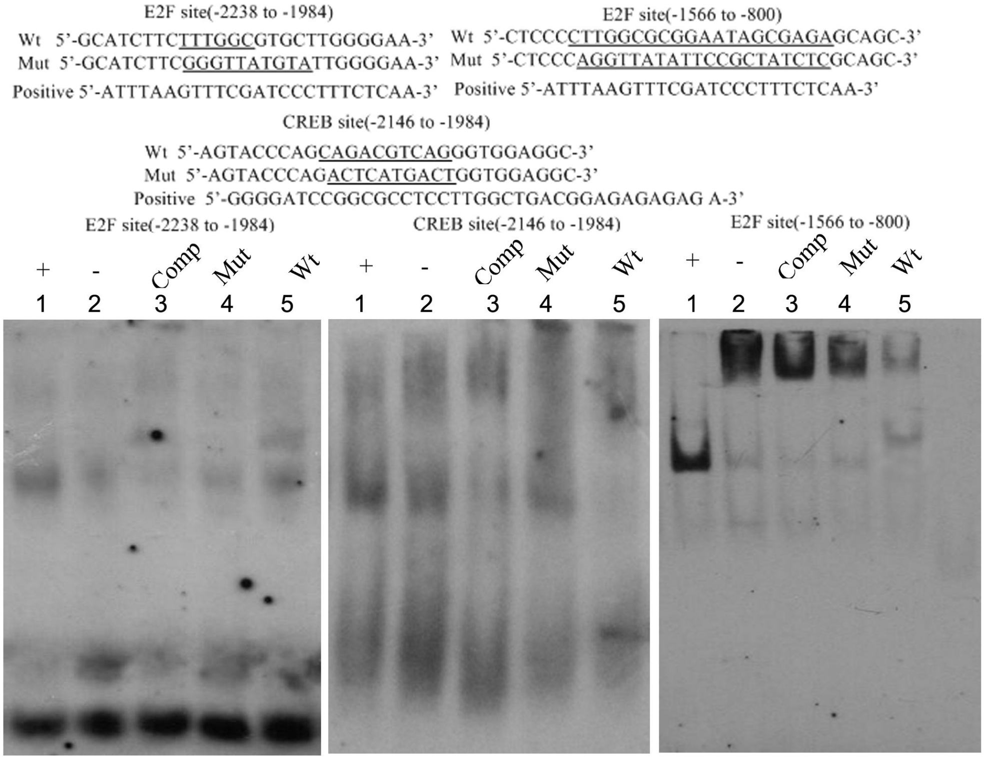

Fig.S6. Identification of transcription factors bound to miR-148a promoter by EMSA. EMSA analysis performed with extracts of hMSCs-ad with the CREB/E2F binding site. Nuclear proteins extracted from hMSCs-Ad were incubated with

digoxigenin-ddUTP-labeled CREB probe. The probe of digoxigenin-ddUTP-labeled CREB/E2F was incubated with cancer cell in lane 1 as a positive (+). The probe of digoxigenin-ddUTP-labeled CREB/E2F was incubated with nuclear proteins extracted from hMSCs-Ad in lane 2 was an absent (−). 50-fold excess of unlabelled cold competitors (Comp; lane3), 50-fold excess of unlabelled mutated CREB/E2F (Mut; lane 4), the probe of digoxigenin-ddUTP-labeled wild type CREB/E2F (Wt; lane5). Sequences of the double-stranded DNA probe containing the predicted Wt CREB (nt -2146 to nt -1984), E2F (nt -2238 to nt -1984, nt -1566 to nt -800) binding domain, positive and the mutation oligonucleotides (Mut) are used in EMSA analysis. + = positive; - = absent; Comp = competitor.

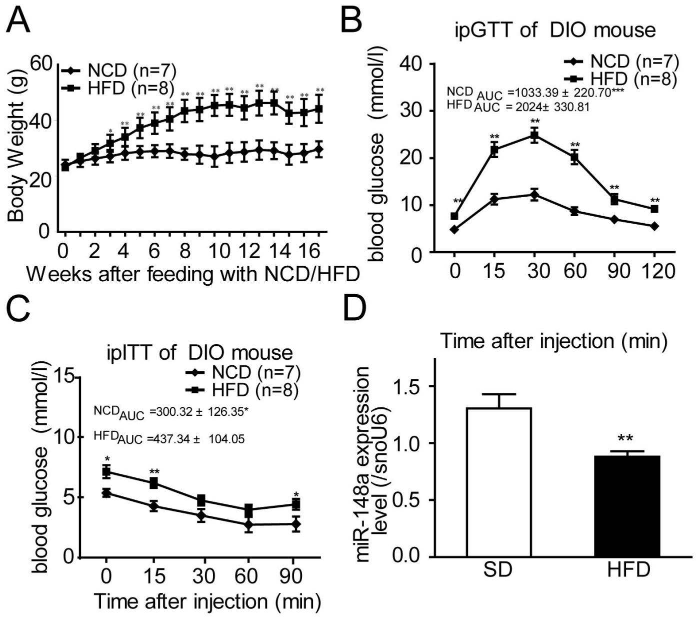

Fig.S7. High-fat diet (HFD) affects weight gain and insulin resistance in mice. (A) the Body weight was measured in two groups of C57BL/6J mice. One group fed a standard diet (SD), and the other group fed a HFD for 17 weeks. Body weight of C57BL/6J mice fed a standard diet (SD) and a HFD for 1–17 weeks (*n*=10 per point of each group). (B) Glucose tolerance test on both groups of mice after 16 h fasting (*n* = 7 mice in SD, *n* = 8 mice in HFD). Blood samples were collected at various time points (0, 15, 30, 60, 90, and120 min) after glucose administration. After 17 weeks of HFD, the mice became more insulin resistant and less glucose tolerant than the SD

group. Glucose and insulin tolerance tests were performed after 17 weeks of SD or HFD feeding. (D) miR-148a expression levels in the brown fat pads of SD or HFD mice were determined by qRT-PCR. A–E: Data represent the mean ± SEM. *$P < 0.05$, **$P < 0.01$ compared with SD mice (Student's *t*-test, paired, two-tailed). SD = standard diet; HFD = high fat diet; ipGTT = intraperitoneal glucose tolerance test; ipITT = intraperitoneal insulin tolerance test.

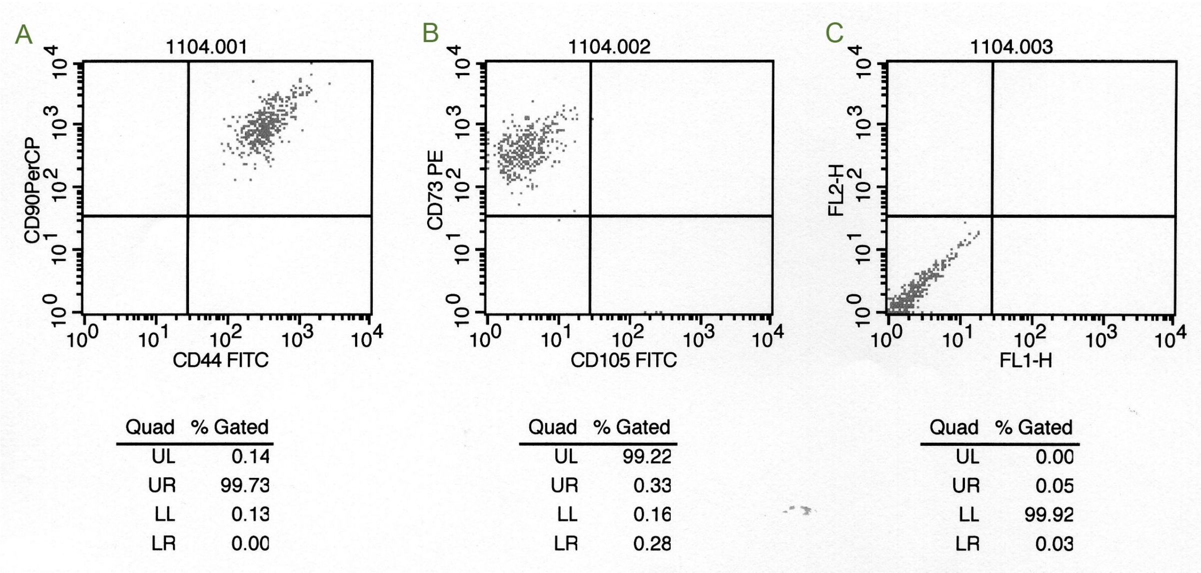

Fig. S8. Human adipose-derived mesenchymal stem cells were characterized by Flow cytometry with antibodies specific to CD44, CD90.and CD73. (A) hMSCs-Ad were characterized by flow cytometry with antibodies specific to CD90 and CD44. (B) hMSCs-Ad were characterized by flow cytometry with antibodies specific to CD73 and CD105. (C) hMSCs-Ad were characterized by flow cytometry without antibody.

Supplemental Table 1. Characteristics of the study subjects.

	Subject	Age	Gender	Height (m)	Weight (kg)	BMI
Normal	1	31	Female	1.56	43.12	17.72
	2	44	Female	1.58	49.70	19.91
	3	48	Female	1.55	53.22	22.15
	4	41	Female	1.62	50.23	19.14
	5	69	Male	1.75	60.03	19.60
	6	50	Female	1.59	51.07	20.20
	7	79	Female	1.56	50.64	20.81
	8	69	Male	1.57	51.52	20.90
	9	54	Female	1.59	54.63	21.61
	10	73	Male	1.79	70.39	21.97
	11	44	Female	1.55	53.36	22.21
	12	27	Male	1.73	67.31	22.49
	13	51	Female	1.54	53.74	22.66
	14	48	Female	1.58	56.59	22.67
	15	37	Female	1.63	61.00	22.96
	16	49	Female	1.56	55.88	22.96
Overweight	17	43	Female	1.58	59.41	23.80
	18	44	Female	1.61	61.77	23.83
	19	57	Female	1.60	62.31	24.34
	20	51	Female	1.61	65.42	25.24
	21	42	Female	1.56	61.79	25.39
	22	67	Male	1.72	77.69	26.26
	23	49	Female	1.57	65.74	26.67
	24	47	Male	1.78	76.14	24.03
	25	45	Female	1.61	64.39	24.84
	26	46	Female	1.59	61.66	24.39
	27	41	Female	1.57	67.39	27.34
	28	60	Female	1.63	73.68	27.73
	29	41	Female	1.54	68.92	29.06
	30	72	Female	1.63	79.97	30.10

Supplemental Table 2. Primers for PCR cloning

Name	Product size (bp)	Reverse and forward primers (5′–3′)
Pro-148a1(for pTB-Cherry)	1734	F: 5′- *GAG*ATGCATTCTGCCCTGCAGCAGCTT-3′<br>R:5′- *CATG*CTCGAGTCAAAAGACCAAACGTGCTGTC-3′
Pro-148a2(for pTB-Cherry)	1239	F:5′-*GGAG*ATGCATCTCCGAAGCAAACAATGAAA -3′<br>R: 5′- *CATG*CTCGAGGTGCGAAATGGAAACCTC -3′
Pro-miR148a1 (for pGL3-Basic)	1503	F: 5′-*GCGC*CTCGAG TCTGCCCTGCAGCAGCTT-3′<br>R: 5′-*CCG*AAGCTTTTCAAAAGACCAAACGTGCTGTC-3′
Pro-miR148a2 (for pGL3-Basic)	1239	F: 5′-*GCGCCTCGAG*CTCCGAAGCAAACAATGAAA-3′<br>R: 5′- *CCGAAGCTT* GTGCGAAATGGAAACCTC -3′
Pro-miR148a2-CREB (for pGL3-Basic)	286	F:5′-*GCGC*CTCGAGCTCCGAAGCAAACAATGAA-3′<br>R: 5′- *CCG* AAGCTT GTCTCCTCCAGCCCCCCA -3′
Pro-miR148a2-CREB2 (for pGL3-Basic)	254	F:5′-*GCGC*CTCGAG GAGAGGGAAGGAAGGAAG -3′<br>R: 5′- *CCG* AAGCTT AGGCCGGTTTGCCTCCAC -3′
Pro-miR148a1-CEBP (for pGL3-Basic)	176	F:5′-*GCGC*CTCGAGTCTGCCCTGCAGCAGCTT-3′<br>R: 5′- *CCG*AAGCTT CGCTGTCCCCGGCCAGCC -3′
Pro-miR148a1-E2F (for pGL3-Basic)	1261	F:5′-*GCGC*CTCGAGCCCGGCCCGAGTGTCGCG-3′<br>R: 5′- *CCG*AAGCTTTGGTCGAACCCTCACCTC -3′
Wnt1 (for pSi-CHECK™ -2)	638	F: 5′- *GCGC*CTCGAGGTTCATACGCATCCCATCT -3′<br>R: 5′- *ATTT*GCGGCCGCGGACCCAGCACAATAAATAG -3′
Wnt10b (for pSi-CHECK™ -2)	430	F: 5′-*GCGC*CTCGAG CCCTTTGCTCTGATTTCCTTCC-3′<br>R: 5′- *ATTT*GCGGCCGC TGTCTCCCATTATCCCACA -3′
E2F3 (for pSi-CHECK™ -2)	796	F: 5′- *GCGC*CTCGAGGTAGGCACATAGTAGGCA -3′<br>R: 5′-*ATTTG*CGGCCGCCTTAAAGTAACGCAAATAC -3′
DNMT1 (for pSi-CHECK™ -2)	261	F: 5′- *GCGC*CTCGAGCCCTCCCGTCACCCCTGTT -3′<br>R: 5′- *ATTTG*CGGCCGCAAGTCTTAATTTCCACTC-3′
DNMT3B (for pSi-CHECK™ -2)	558	F: 5′- *GCGC*CTCGAGTAACAACGGCAAAGACCG -3′<br>R: 5′- *ATTT*GCGGCCGCCCTACCTTTATGCCCAACTC -3′

Underlined bases are restriction sites; *italic* bases are protected bases.

Supplemental TableE 3. Primers for qRT-PCR

name	Reverse and forward primers (5′–3′)	probe
PPARγ2	F:5′-AAATATCAGTGTGAATTACAGCAAACC-3′<br>R: 5′- GGAATCGCTTTCTGGGTCAA -3′	5′-TGCTGTTATGGGTGAAACTCTGGGAGATTCT-3′
CEBP	F: 5′-GGGTCTCTAGTTCCACGCCT-3′<br>R: 5′-CCCATCGCAGTGAGTTCCG-3′	5′-TCCCACCTCCCTCCGCACACACC-3′
FABP4	F: 5′-GGTGGTGGAATGCGTCATG-3′<br>R:5′-CAACGTCCCTTGGCTTATGC-3′	5′-AAGGCGTCACTTCCACGAGAGTTTATGAGA-3′
Wnt1	F:5′-CAGCGACAACATTGACTTCG-3′<br>R:5′-GGCGCATCTCGGAGAATA-3′	Roche # 7
18s	F: 5′-CGGGTCGGGAGTGGGTAAT-3′<br>R: 5′-AGTCGCCGTGCCTACCAT-3′	CGCCTGCTGCCTTCCTTGGATGTG

Supplemental Table 4. Primers for qChiP-PCR

	Reverse and forward primers (5′–3′)
CREB binding sites	F:5′-GATGTACACAACGGTATGCA-3′
	R: 5′-CCCGCTCTGCTGCCTGTTCT-3′
E2F binding sites	F:5′-CTCCGAGCCCCAGCAAGCAC-3′
	R: 5′-TTCTTCCCATCCACTCTTCT-3′

Supplemental Blots original data

Fig.2F PPAR γ2

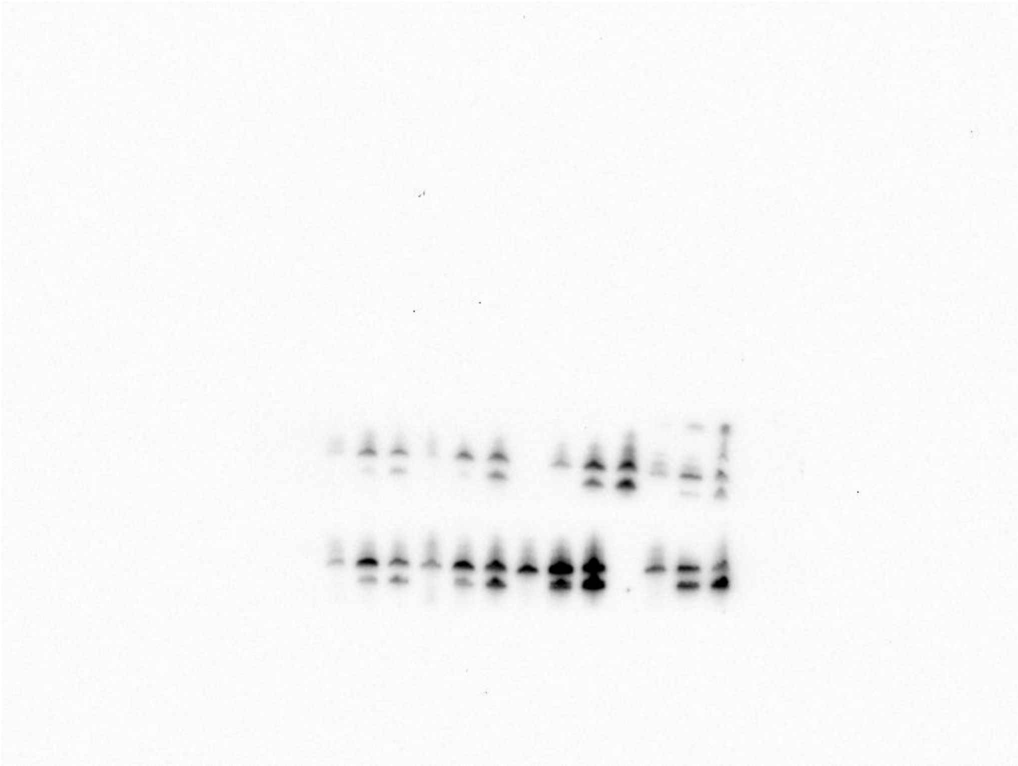

Lanes order：first 6 lanes in the last line are the target lanes that were marked in the follow picture.

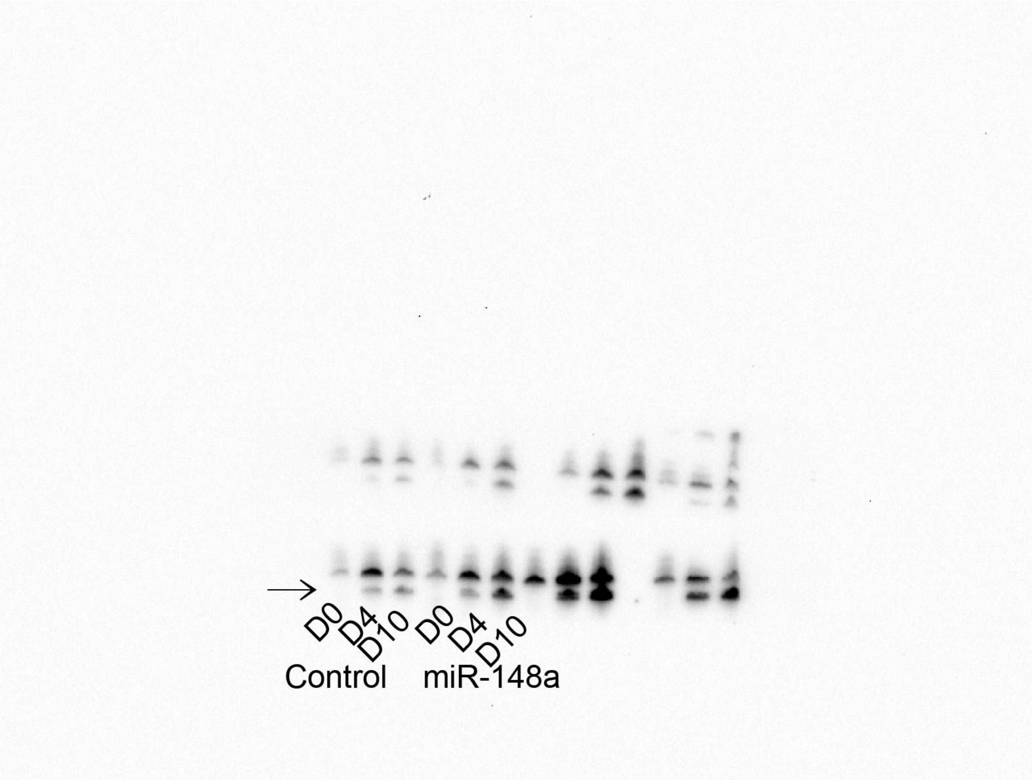

Fig.2F CEBP-α

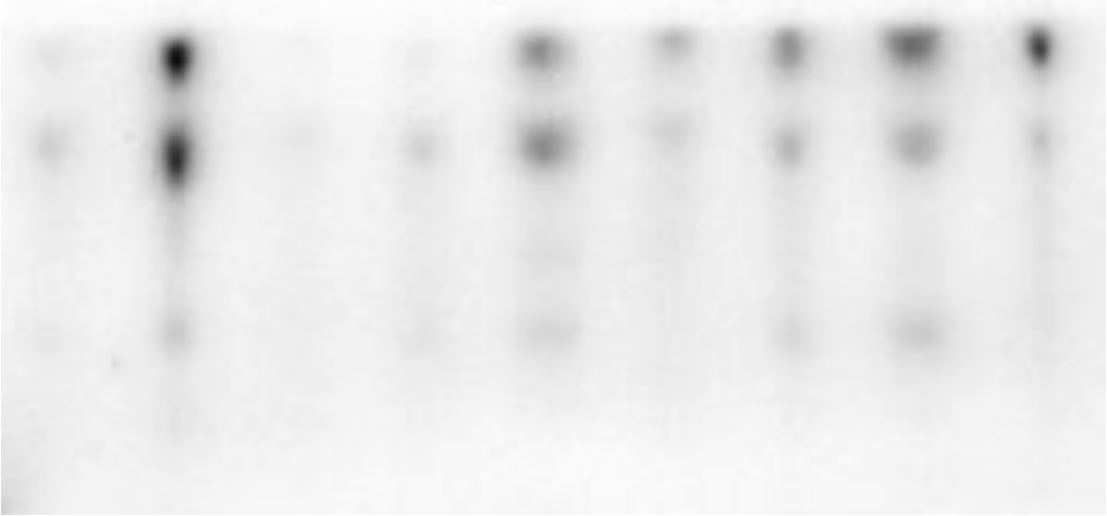

Lanes order：last 6 lanes in the first line are the target lanes that were marked in the follow picture.

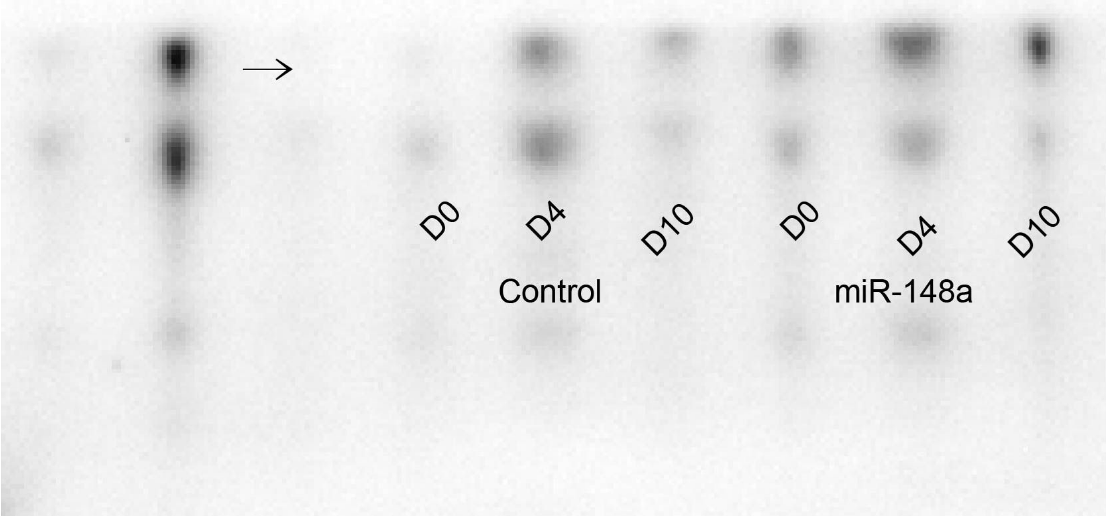

Fig.2F GAPDH

Original

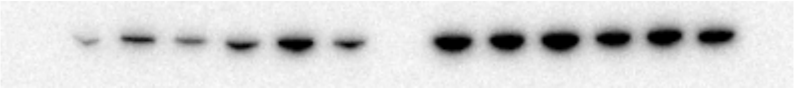

Makerd

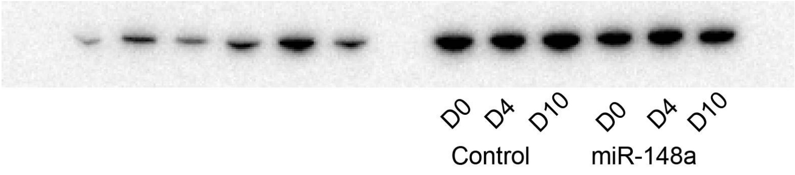

Fig.5A Wnt1

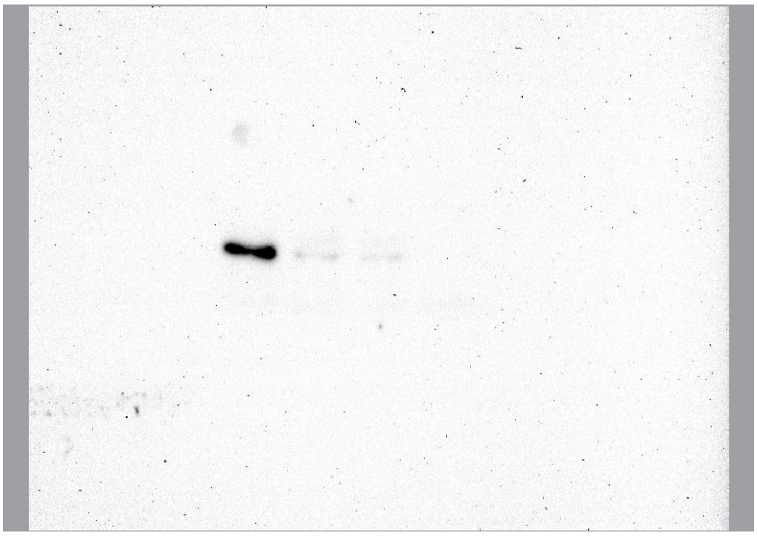

Fig.5A P-GSK-3β

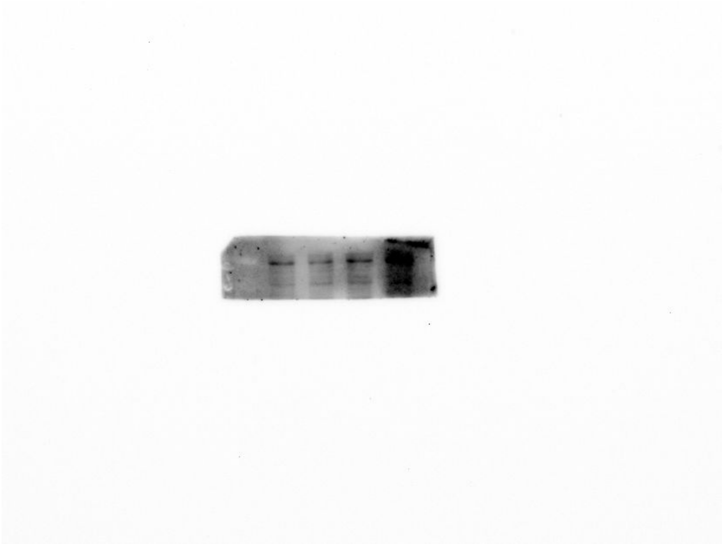

Fig.5A GSK-3β

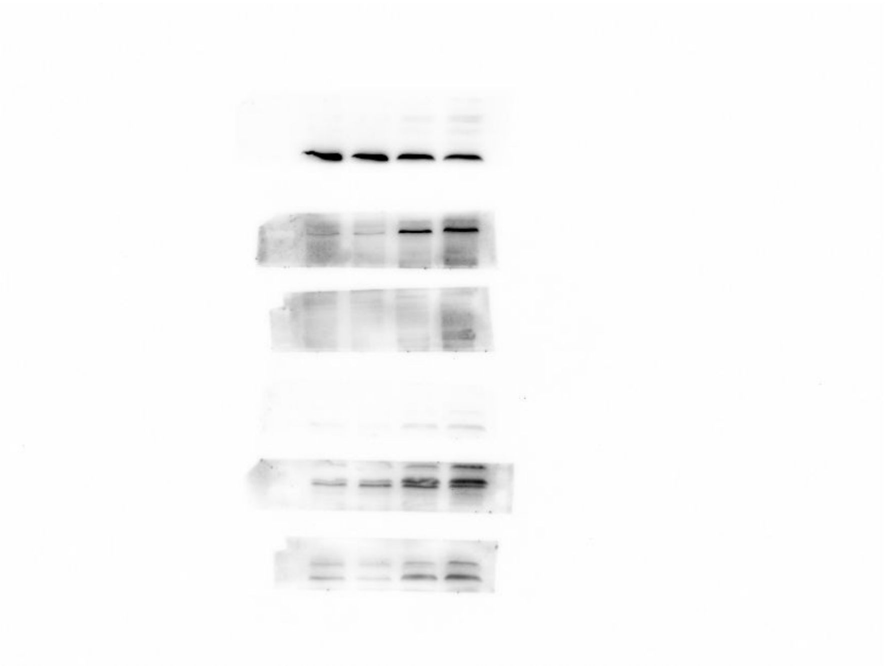

Lanes order：first line is the target lane that was marked in the follow picture.

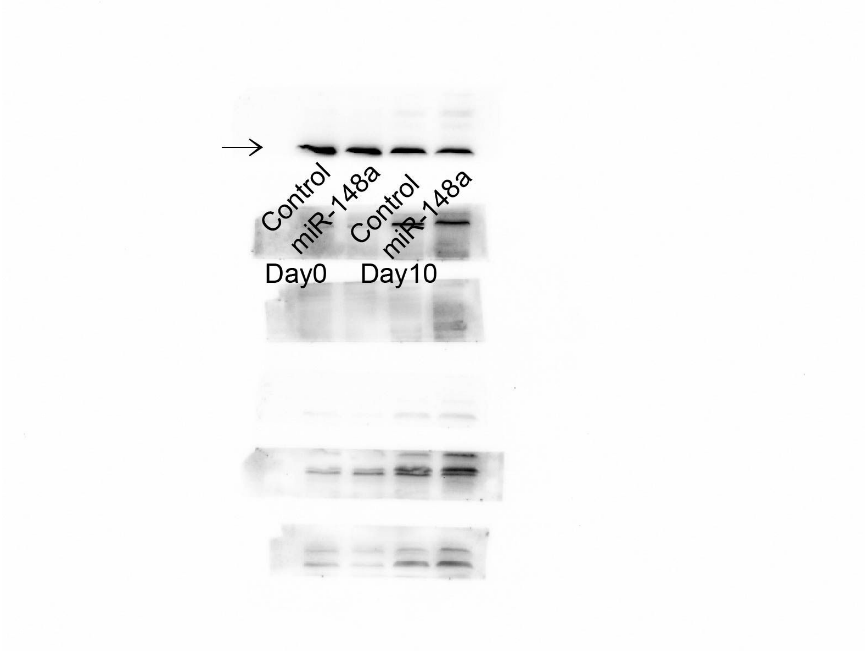

Fig.5A　Cyto-β-catenin

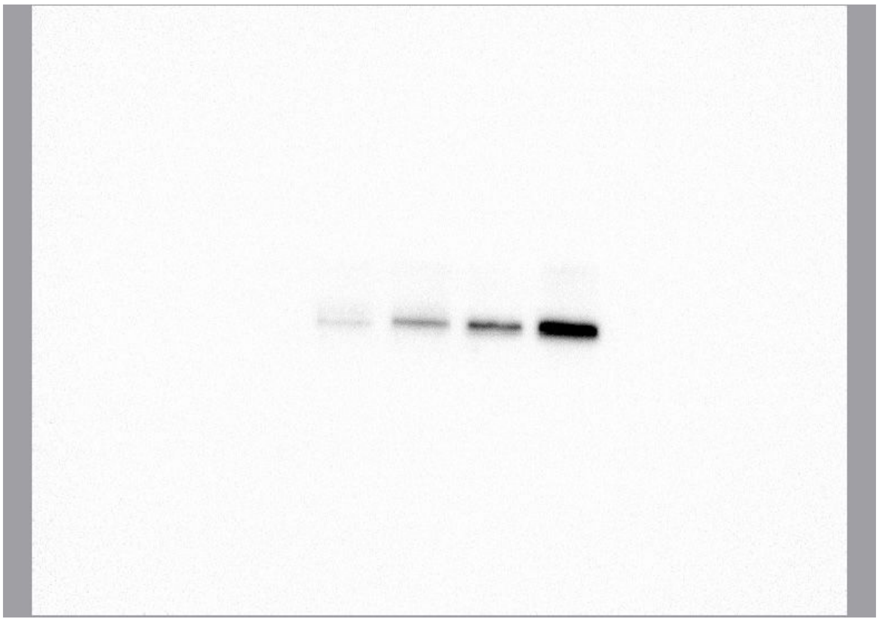

Fig.5A nuclear-β-catenin

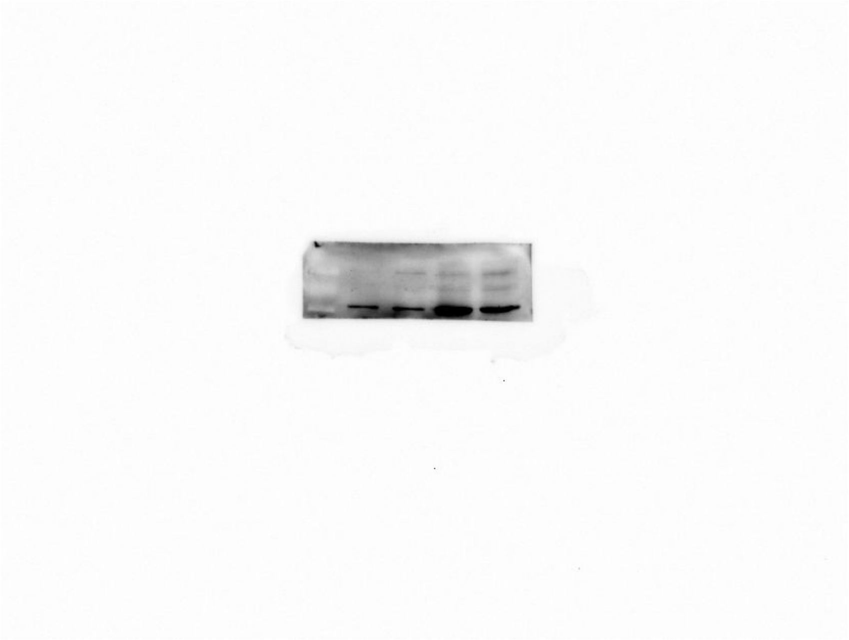

Fig.5A GAPDH

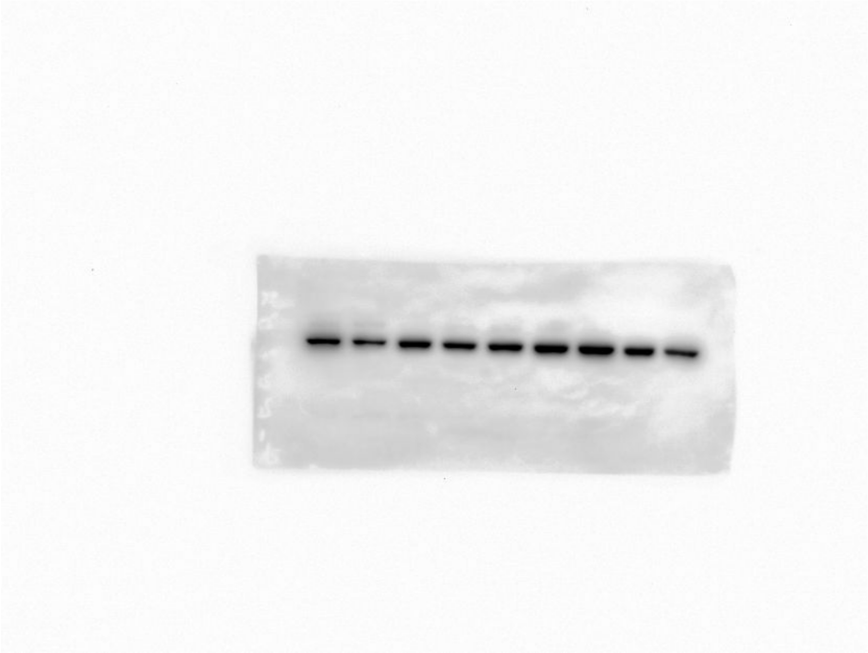

Lanes order：first 4 lines are the target lanes that were marked in the follow picture.

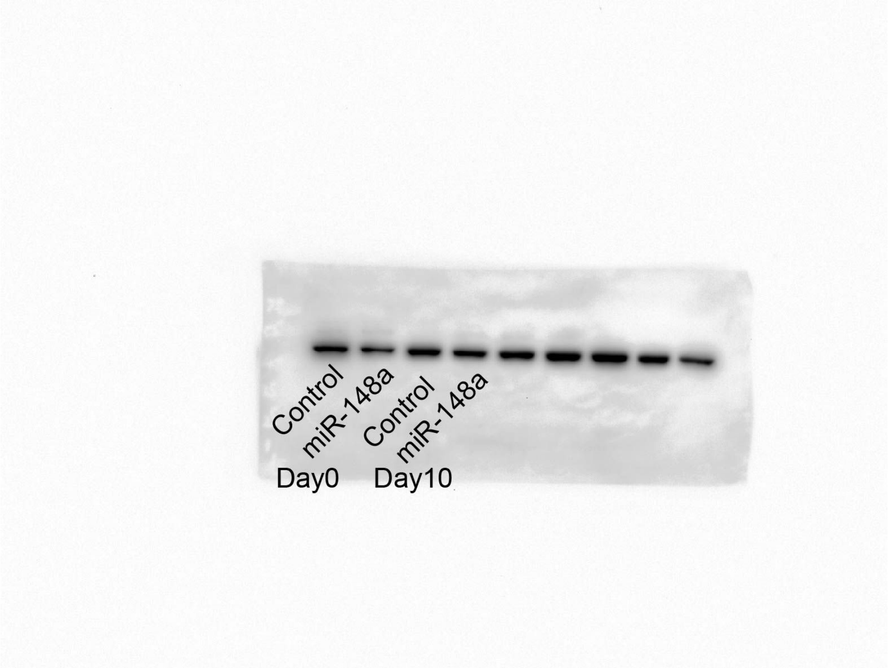

Fig.5B Wnt silence

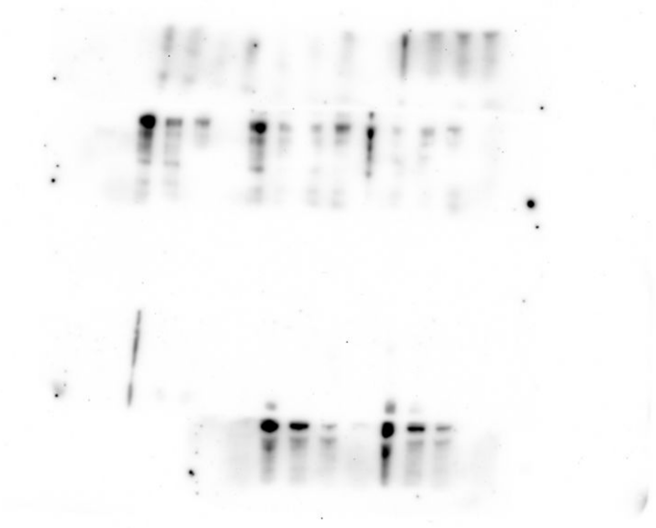

Lanes order：first 3 lines are the target lanes in the last line that were marked in the follow picture.

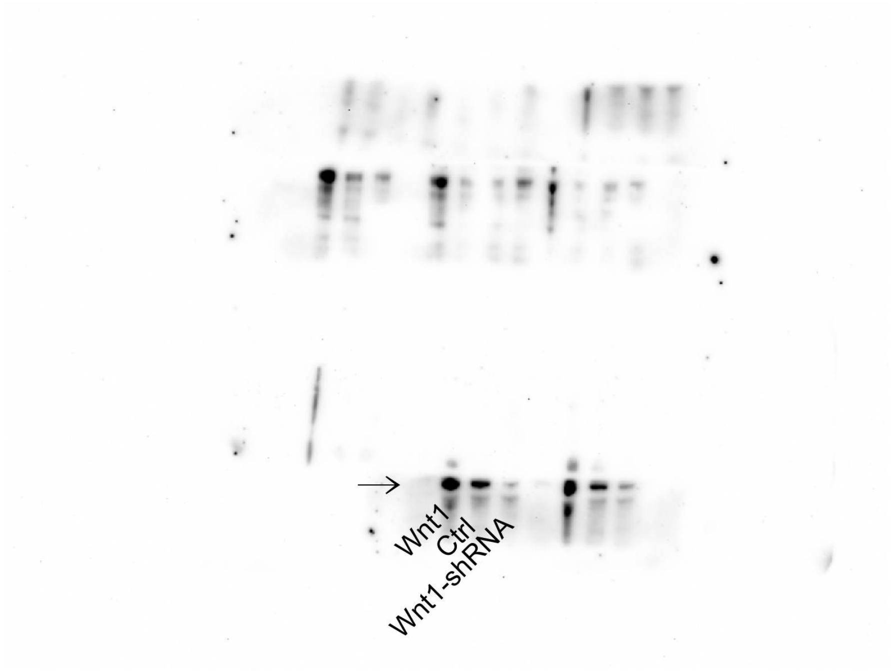

Fig.5B GAPDH

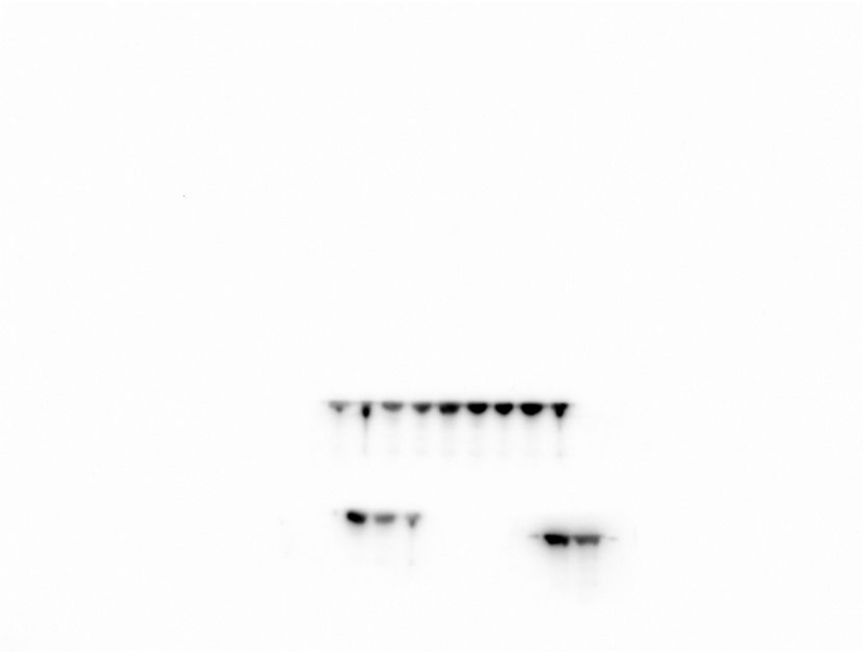

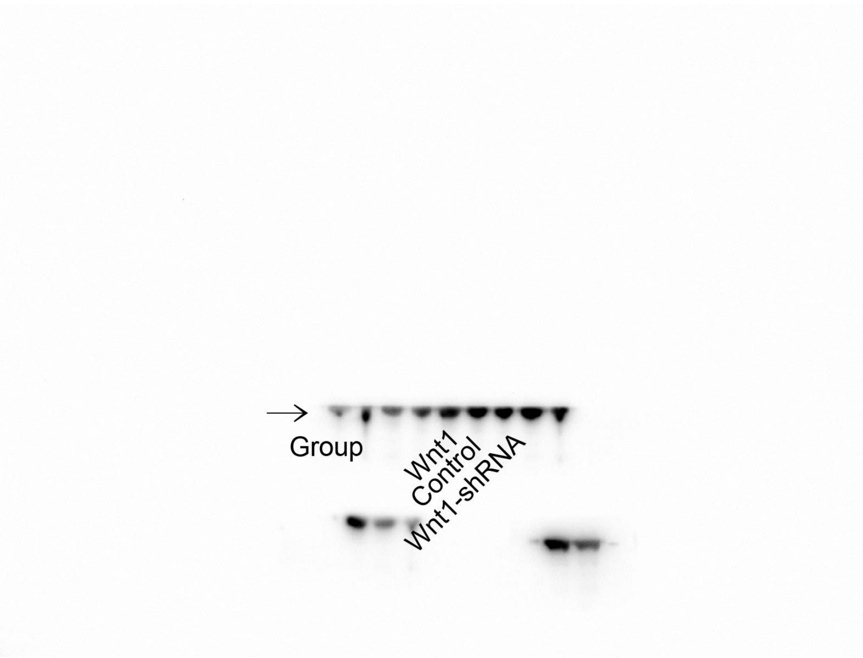
Group
Wnt1
Control
Wnt1-shRNA
